# The efficacy and safety of ceftazidime/avibactam or polymyxin B based regimens for *carbapenem-resistant Pseudomonas aeruginosa* infection: a multicenter real-world and propensity score-matched study

**DOI:** 10.3389/fphar.2025.1533952

**Published:** 2025-03-31

**Authors:** Wen-Ming Long, Wei-Xin Xu, Qin Hu, Qiang Qu, Xiao-Li Wu, Ying Chen, Qing Wan, Tian-Tian Xu, Yue Luo, Jian Qu

**Affiliations:** ^1^ Department of Pharmacy, The Second Xiangya Hospital, Central South University, Institute of Clinical Pharmacy, Central South University, Changsha, China; ^2^ Department of Pharmacy, The Second People’s Hospital of Huaihua City (The Central Hospital of Huaihua City), Huaihua, China; ^3^ Department of Pharmacy, Xiangya Hospital, Central South University, Changsha, China; ^4^ Department of Pharmacy, The Second Affiliated Hospital of Guangzhou Medical University, Guangzhou, China; ^5^ Department of Pharmacy, Renmin Hospital, Wuhan University, Wuhan, China; ^6^ Department of Pharmacy, The First Affiliated Hospital of Nanchang University, Nanchang, China; ^7^ Department of Pharmacy, The People’s Hospital of Liuyang, Liuyang, China; ^8^ Hunan Key Laboratory of the Research and Development of Novel Pharmaceutical Preparations, Changsha Medical University, Changsha, China

**Keywords:** ceftazidime/avibactam, polymyxin B, carbapenem-resistant *Pseudomonas aeruginosa*, real-world study, propensity score-matched, microbiological efficacy

## Abstract

**Introduction:**

*Carbapenem-resistant Pseudomonas aeruginosa* (*CRPA*) infections pose a critical clinical challenge. Although ceftazidime/avibactam (CAZ/AVI) and polymyxin B (PMB) are frontline therapies, their comparative effectiveness in terms of 30-day survival, renal safety profiles, and clinical success rates remains poorly characterized. To address this knowledge gap, a multicenter real-world study was conducted.

**Methods:**

*CRPA*-infected patients treated with PMB or CAZ/AVI-based regimens were enrolled from five hospitals between January 1, 2021, to July 31, 2023. Propensity score matching (PSM) and binary logistic regression analysis were performed to evaluate efficacy and acute renal injury (AKI) occurrence, and a multivariable COX proportional hazards regression of the 30-day all-cause mortality was performed.

**Results:**

170 *CRPA*-infected patients were enrolled, among whom 124 (72.9%) had d*ifficult-to-treat resistant P. aeruginosa (DTR-PA)* infections and 77 (45.3%) received CAZ/AVI-based regimens. After 1:1 PSM, the results demonstrated that the *CRPA* clearance rate was significantly higher in the CAZ/AVI group compared to the PMB group (61.0% vs. 24.4%, *p* = 0.001); however, no significant differences were observed in clinical success rates (55.6% vs. 44.4%), incidence of AKI (26.8% vs. 39.0%), or 30-day all-cause mortality (7.3% vs. 12.2%) between the two groups (all *p* > 0.05). Compared with the PMB-based regimens, CAZ/AVI-based regimens were significantly associated with *CRPA* clearance success (OR 0.185, 95%CI 0.061–0.564, *p* < 0.001); additionally, multi-site infection (OR 0.295, 95%CI 0.097–0.899, *p* = 0.032) and the number of combined anti-*PA* antibiotics (OR 0.435, 95%CI 0.213–0.888, *p* = 0.022) were associated with enhanced *CRPA* clearance. The occurrence of AKI in patients with *CRPA* infection was associated with underlying diseases, including sepsis/septic shock (OR 3.405, 95%CI 1.007–11.520, *p* = 0.049), and diabetes mellitus (OR 3.600, 95%CI 1.018–12.733, *p* = 0.047). In addition, other *CREs* infection (HR 40.849, 95%CI 3.323–502.170, *p* = 0.004), APACHE II score (HR 1.072, 95%CI 1.032–1.114, *p* < 0.001) were found to be independent predictors of 30-day all-cause mortality.

**Conclusion:**

In conclusion, CAZ/AVI-based regimens demonstrated superior efficacy in clearing *CRPA* compared to PMB-based regimens. Furthermore, several factors associated with AKI and mortality in *CRPA*-infected patients were identified, highlighting the need for further research to optimize treatment strategies.

## 1 Introduction

The escalating prevalence of multidrug-resistant organisms (MDROs) poses a significant threat to global public health. In critical infections, particularly within intensive care units (ICUs), the identification of causative pathogens is exceptionally challenging due to infection complexity and potential polymicrobial involvement. Among these, *Pseudomonas aeruginosa (PA)* represents one of the paramount concerns (WHO, 2024; Borgatta et al., 2017). As a common pathogen with multifaceted resistance mechanisms, *PA* is recognized as one of the six most lethal multidrug-resistant pathogens under the *ESKAPE* classification (*Enterococcus faecium, Staphylococcus aureus, Klebsiella pneumoniae, Acinetobacter baumannii, P. aeruginosa,* and *Enterobacter* spp.) (Antimicrobial, 2022).

Since in clinical practice it is difficult to ascertain the multiple resistance mechanisms present in every *PA* strain, and particularly carbapenem-resistance, the concept of difficult to treat resistant *PA* (*DTR-PA*) has emerged (Cosentino et al., 2023). Data from the CHINET surveillance system (http://www.chinets.com) revealed that in 2023, *PA* accounted for 7.8% of 445,199 bacterial isolates collected through active surveillance across 74 tertiary hospitals in China. Notably, 17.4% and 21.9% of these *PA* isolates exhibited resistance to meropenem and imipenem, respectively. Alarmingly, the prevalence of carbapenemase production in carbapenem-resistant *PA* (*CRPA*) in China has reached 41% (Zhang et al., 2023), with approximately 34%–38% of *CRPA* strains classified as *DTR-PA* (Dong et al., 2025; Yuan et al., 2023). The attributable mortality of *CRPA* infections is estimated between 20.0% and 30.8% (Lodise et al., 2022), while mortality associated with *DTR-PA* may escalate to 43% (Yuan et al., 2023).

Current therapeutic options for *CRPA* infections remain severely limited (Reig et al., 2022). Ceftazidime/avibactam (CAZ/AVI) is considered as one of the first-line treatment for *CRPA* infections, whereas polymyxin B (PMB) is reserved as a last-resort therapy (Pulmonary, 2022; Tamma et al., 2024). However, emerging reports of *CRPA* resistance to both PMB and CAZ/AVI, coupled with the nephrotoxicity associated with these agents, have constrained their clinical utility (Howard-Anderson et al., 2022; Wang et al., 2025; Shi et al., 2024; Chang et al., 2022). Specifically, PMB and CAZ/AVI are linked to drug-related renal insufficiency, which complicates dosing regimens and exacerbates treatment failure risks. This highlights the critical need to elucidate the real-world clinical efficacy and renal safety profiles of CAZ/AVI-based and PMB-based regimens in the treatment of CRPA infections, with a focus on comprehensive clinical considerations.

While some small-sample studies have been conducted, they have provided limited insights. For instance, Xu et al. reported a clinical cure rate of 63.1% for CAZ/AVI in treating *CRPA*, but their study did not conclusively determine whether monotherapy or combination therapy was more effective, as no significant difference in clinical efficacy was found between the two approaches (Xu et al., 2024). Additionally, a single-center retrospective cohort study (Chen et al., 2022) compared PMB and CAZ/AVI in *CRPA* treatment and found that CAZ/AVI seemed to offer better survival benefits than PMB, with age, CAZ/AVI use, and central venous catheter placement identified as independent predictors of 30-day survival. However, this study had notable limitations, as it did not evaluate the safety of PMB and CAZ/AVI, and the assessment of their microbiological efficacy was not comprehensive. The existing literature thus falls short in providing a complete picture of the comparative effectiveness and safety of these treatments, as well as the factors influencing their outcomes. This multicenter retrospective cohort study aims to compare the clinical effectiveness of PMB and CAZ/AVI in treating *CRPA* infections, with specific emphasis on evaluating the impact of antibiotic treatment regimens, *DTR-PA* infections, microbiological clearance rates, and AKI incidence on 30-day all-cause mortality, while systematically identifying associated clinical influencing factors.

## 2 Materials and methods

### 2.1 Study setting and participating centers

This multicenter retrospective cohort study, conducted from January 2021 to July 2023 at five tertiary hospitals in China (Second Xiangya Hospital (3,500 beds), Xiangya Hospital (3,500 beds), Second Affiliated Hospital of Guangzhou Medical University (2,500 beds), First Affiliated Hospital of Nanchang University (6,000 beds), and Renmin Hospital of Wuhan University (3,500 beds)). Hospital selection criteria included: (1) Provincial-level tertiary centers with >2000 beds; (2) Established antimicrobial stewardship programs; (3) Complete electronic medical record systems covering ICU and general wards.

### 2.2 Ethics

The study was conducted in accordance with the ethical standards outlined in the Helsinki Declaration (1964). Approval was obtained from the Ethics Committees of the Second Xiangya Hospital of Central South University (LYF-2020021) and other ethicscommittees at each study site. Given the retrospective and observational design of the study, the requirement for written informed consent was waived.

### 2.3 Patients

*CRPA* infection in a patient was defined as the detection of *CRPA* accompanied by a body temperature >38.3°C or <36°C, along with a white blood cell count >12 × 10^9^/L or <4 × 10^9^/L, C-reactive protein (CRP) > 50 mg/L (measured by immunoturbidimetry) or procalcitonin (PCT) ≥0.5 ng/mL (measured by electrochemiluminescence), new onset of purulent sputum or changes in sputum characteristics, and progression of infiltrates on chest imaging within 72 h. Inclusion criteria of patients were: (1) patients confirmed to have *CRPA* infection by bacterial culture and sensitivity testing; (2) patients treated with PMB or CAZ/AVI-based therapy for ≥72 h; (3) patients with infection-related indicators (Body temperature, white blood cell count, neutrophil count, CRP, and PCT) to assess treatment efficacy. Exclusion criteria were: (1) age <18 years; (2) pregnant patients; (3) patients unable to assess efficacy; (4) PMB maintenance dose <50 mg q12h; (5) cases of resistance of *CRPA* to CAZ/AVI or PMB; (6) Patients for whom the microbiological efficacy could not be determined at the end of treatment due to the irregular re-examination of pathogens.

### 2.4 Collection of clinical data

Demographic characteristics, clinical features, microbiological data, etc., including age, weight, comorbidities, Acute Physiology and Chronic Health Evaluation II (APACHE II) score, site of infection, pathogens causing infection, details of antibiotic use, and inflammatory markers, were extracted from the hospital electronic medical record system. The primary outcome of interest was 30-day all-cause mortality, with secondary outcomes including microbiological clearance, clinical efficacy, and AKI.

### 2.5 Microbiological identification

All isolates were identified by MALDI-TOF MS (bioMérieux) with ≥98.7% confidence. Antimicrobial susceptibility testing utilized VITEK^®^2 platforms, supplemented by CLSI M07-compliant broth microdilution for CAZ/AVI. Minimum inhibitory concentration (MIC) interpretations uniformly applied CLSI M100-Ed33 (2023) criteria, except where unavailable: EUCAST ECOFFs (v13.0) guided PMB interpretation. Historical MIC data were reanalyzed using 2023 standards to eliminate temporal guideline discrepancies, adhering to China’s WS/T 639–2018 mandate prioritizing CLSI. *CRPA* required meropenem MIC ≥8 mg/L (CLSI 2023). Given the real-world retrospective design focusing on *CRPA*, systematic β-lactamase/carbapenemase phenotypic testing was not performed. *DTR-PA* was defined as *PA* exhibiting non-susceptibility to all of the following: piperacillin-tazobactam, ceftazidime, cefepime, aztreonam, meropenem, imipenem-cilastatin, ciprofloxacin, and levofloxacin (Tamma et al., 2024).

### 2.6 Outcome measures and definitions

#### 2.6.1 Microbiological efficacy

Patients with *CRPA* infection were treated with PMB or CAZ/AVI-based regimens, focusing on single *CRPA* strains. Microbiological clearance group: all infection sites sampled for microbial culture after treatment with PMB or CAZ/AVI were negative of *CRPA*.

#### 2.6.2 Clinical efficacy

Patients with *CRPA* infection treated with PMB or CAZ/AVI-based regimens. Clinical efficacy group: hemodynamically stable without the need for vasopressors, body temperature <37.5°C for 72 h, white blood cell count <10 × 10^9^/L; improvement in clinical symptoms, infection indicators (CRP, PCT), and microbiological indicators. Clinical inefficacy group: did not meet any criteria of the clinical efficacy group, worsened condition leading to treatment discontinuation, or cases resulting in hospital mortality.

#### 2.6.3 Acute kidney injury--based on the KDIGO criteria

After completing treatment with PMB or CAZ/AVI-based regimens, creatinine levels should be monitored. Changes in renal function were categorized according to the KDIGO classification. The AKI group was defined as an increase in creatinine levels by either 26.5 μmol/L (observed within 48 h of CAZ/AVI or PMB administration) or 1.5 times the baseline level by the end of treatment. Due to the retrospective nature of this study, we were unable to observe changes in urine output.

#### 2.6.4 30-Day all-cause mortality

Patients with *CRPA* infection treated with PMB or CAZ/AVI-based regimens. Mortality group: all-cause mortality or treatment discontinuation due to worsened condition within 30 days after PMB or CAZ/AVI treatment.

### 2.7 Statistical methods

Statistical analyses were conducted using SPSS version 25.0. Continuous variables were summarized as mean ± standard deviation for normally distributed data or median with interquartile range (IQR) for non-normally distributed data. Comparisons between groups for continuous variables were performed using independent samples t-tests for normally distributed data and non-parametric tests (e.g., Mann-Whitney U test) for non-normally distributed data. Categorical variables were presented as numbers and percentages and analyzed using chi-square tests or Fisher’s exact tests, as appropriate. Propensity score matching (PSM) was performed in a 1:1 ratio, incorporating variables with *p* < 0.1 in the univariate analysis of CAZ/AVI and PMB, as well as covariates influencing the matched cohort. The matching tolerance was set at 0.2, and the order of cases was randomly permuted during the matching process to minimize selection bias. Treatment outcomes, including therapeutic efficacy, microbiological clearance rate, mortality, and AKI, were analyzed using chi-square tests or Fisher’s exact tests before and after PSM. Survival time and time to microbiological clearance were analyzed using non-parametric tests (e.g., Kaplan-Meier analysis with log-rank test). In the analysis of the impact of different administration methods on outcomes, the clinical efficacy, microbial clearance rate, and incidence of AKI were assessed using either the chi-square test or Fisher’s exact test, as appropriate. The 30-day all-cause mortality was evaluated using the log-rank test. In the subgroup analysis of microbiological clearance rates for different treatment regimens, we compared the microbiological clearance rates for monotherapy and combination therapy with CAZ/AVI and PMB before and after PSM. These comparisons were analyzed using chi-square tests or Fisher’s exact tests. For analysis of factors influencing therapeutic efficacy and all-cause mortality, variables with p < 0.05 in the univariate analysis and clinically relevant covariates were included in binary logistic regression or Cox proportional hazards regression models using enter selection. A P value of less than 0.05 was considered statistically significant. During multiple factor analysis, the predicted probabilities from the models were saved and used to construct receiver operating characteristic (ROC) curves using GraphPad software to evaluate the predictive performance on the treatment outcomes.

## 3 Results

### 3.1 Clinical and microbiology characteristics

**FIGURE 1:**
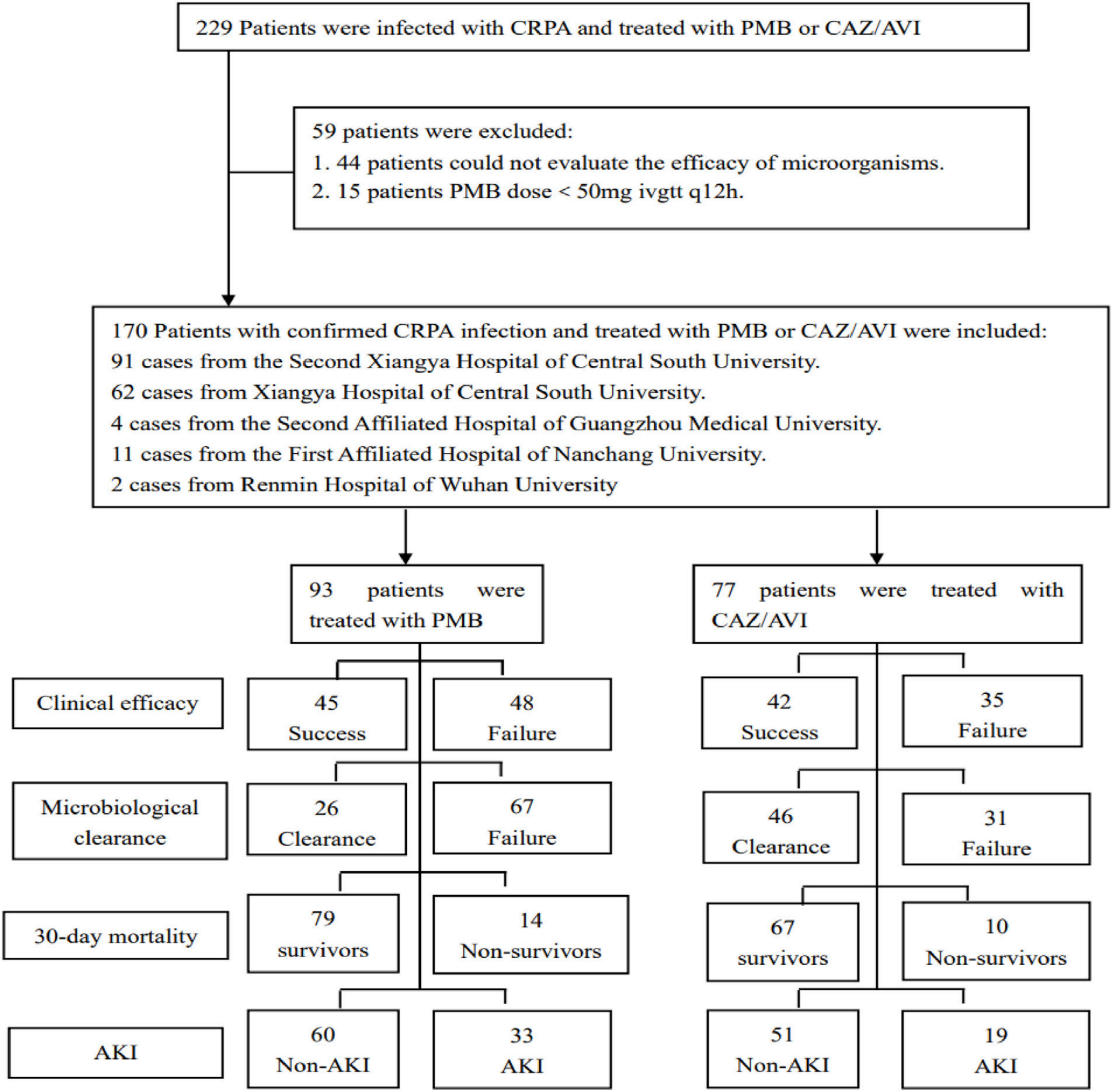
Flow diagram illustrating the process of determining the inclusion and exclusion criteria for patients.

A total of 170 patients infected with *CRPA* and treated with CAZ/AVI or PMB for ≥3 days were included in the study based on the inclusion and exclusion criteria. Among them, 93 (54.7%) cases were treated with PMB-based therapy for *CRPA* infection (Figure 1). The characteristics of the included cases are shown in Table 1. There were 137 (80.6%) male patients, with an average age of 61.2 ± 17.6 years. A total of 44 (25.9%) patients presented with multi-site infections of *CRPA*, while 106 (62.4%) patients were concurrently found to harbor multiple species of *carbapenem-resistant Gram-negative bacteria (CRGNB)*. A total of 124 (72.9%) strains of *CRPA* met the *DTR-PA* criteria. Among the *CRPA* strains, 15 (8.8%) remained sensitive to ceftazidime (Table 2). A combination therapy regimen was employed in 103 (60.6%) patients. The details of PMB and CAZ/AVI use were shown in Supplementary Material S1. Among the patients treated with CAZ/AVI, ten required dose adjustments following the initial administration due to changes in renal function. Specifically, three patients had an increase in the single-dose amount, while seven patients had a decrease in the single-dose amount.

**TABLE 1:** Demographics and clinical characteristics of *CRPA*-infected patients treated with different regimens.

Demographics and clinical characteristics	Before PSM				After PSM			
	Total (N = 170)	PMB (N = 93)	CAZ/AVI (N = 77)	P-value	Total (N = 82)	PMB (N = 41)	CAZ/AVI (N = 41)	P-value
Demographic characteristics								
Age (years)	61.2 ± 17.6	62.0 ± 18.4	60.1 ± 16.7	0.495	61.1 ± 18.3	63.8 ± 20.1	58.3 ± 16.0	0.143
Gender (male)	137 (80.6%)	77 (82.8%)	60 (77.9%)	0.424	66 (80.5%)	36 (87.8%)	30 (73.2%)	0.095
Baseline creatinine (umol/L)	76.5 (49.0–121.3)	78.8 (52.9–117.0)	71.8 (8.0–166.0)	0.971	82.3 (49.2–143.2)	89.4 (54.4–117.0)	74.4 (48.0–175.2)	0.838
Baseline CCR (mL/min)	73.6 (40.0–108.3)	78.6 (42.9–100.9)	72.8 (32.4–116.6)	0.667	74.6 (37.1–102.9)	81.0 (40.9–95.9)	70.2 (29.1–113.1)	0.806
CRRT/RRT	24 (14.1%)	10 (40.8%)	14 (18.2%)	0.166	14 (17.1%)	4 (9.8%)	10 (24.4%)	0.142
Mechanical ventilation	132 (77.6%)	71 (76.3%)	61 (79.2%)	0.654	60 (73.2%)	30 (73.2%)	30 (73.2%)	1.000
Vasoactive drugs	104 (61.2%)	50 (53.8%)	54 (70.1%)	**0.029**	47 (57.3%)	21 (51.2%)	26 (63.4%)	0.264
ICU administration	123 (72.4%)	74 (79.6%)	49 (63.6%)	**0.021**	54 (65.9%)	27 (65.9%)	27 (65.9%)	1.000
Sepsis/septic shock	66 (38.8%)	29 (31.2%)	37 (48.1%)	**0.025**	36 (43.9%)	17 (41.5%)	19 (46.3%)	0.656
Hospital stays (days)	38.5 (24.8–58.8)	36.0 (24.0–60.5)	42.0 (25.5–58.5)	0.585	38.0 (22.0–56.5)	36.0 (29.5–60.5)	42.0 (23.0–55.5)	0.565
APACHE II score	23.0 (20.0–25.3)	23.0 (19.5–23.0)	23.0 (20.0–28.5)	0.452	23.0 (19.0–23.0)	23.0 (19.5–23.0)	21.0 (18.0–23.0)	0.685
Comorbidity								
Solid organ transplantation	6 (3.5%)	2 (2.2%)	4 (5.2%)	0.412	2 (2.4%)	1 (2.4%)	1 (2.4%)	1.000
Hypoproteinemia	52 (30.6%)	18 (19.4%)	34 (44.2%)	**<0.001**	25 (30.5%)	12 (29.3%)	13 (31.7%)	0.810
Respiratory diseases	148 (87.1%)	81 (87.1%)	67 (87.0%)	0.987	68 (82.9%)	35 (85.4%)	33 (80.5%)	0.557
Renal insufficiency	26 (15.3%)	9 (9.7%)	17 (22.1%)	**0.025**	12 (14.6%)	4 (9.8%)	8 (19.5%)	0.349
Diabetes mellitus	42 (24.7%)	17 (18.3%)	25 (32.5%)	**0.033**	22 (26.8%)	10 (24.4%)	12 (29.3%)	0.618
Urinary system disease	26 (15.3%)	14 (15.1%)	12 (15.6%)	0.924	12 (14.6%)	5 (12.2%)	7 (17.1%)	0.532
Digestive system diseases	61 (35.9%)	34 (36.6%)	27 (35.9%)	0.840	34 (41.5%)	20 (48.8%)	14 (34.1%)	0.179
Abnormal liver function	36 (21.2%)	13 (14.0%)	23 (29.9%)	**0.012**	20 (24.4%)	10 (24.4%)	10 (24.4%)	1.000
Cerebrovascular diseases	90 (52.9%)	51 (54.8%)	39 (50.6%)	0.586	39 (47.6%)	23 (56.1%)	16 (39.0%)	0.122
Cardiovascular diseases	97 (57.1%)	42 (45.2%)	55 (71.4%)	**0.001**	48 (58.5%)	23 (56.1%)	25 (61.0%)	0.654
Malignancy	26 (15.3%)	13 (14.0%)	13 (16.9%)	0.600	12 (14.6%)	6 (14.6%)	6 (14.6%)	1.000
*Infection sites*								
Multi-site infection	44 (25.9%)	18 (19.4%)	26 (33.8%)	**0.033**	57 (69.5%)	31 (75.6%)	26 (63.4%)	0.230
Respiratory tract	155 (91.2%)	85 (91.4%)	70 (90.9%)	0.911	76 (92.7%)	37 (90.2%)	39 (95.1%)	0.675
Blood	25 (14.7%)	6 (6.5%)	19 (24.7%)	**0.001**	14 (17.1%)	6 (14.6%)	8 (19.5%)	0.557
Abdominal	17 (10.0%)	6 (6.5%)	11 (14.3%)	0.090	12 (14.6%)	5 (12.2%)	7 (17.1%)	0.532
Urinary tract	9 (5.3%)	4 (4.3%)	5 (6.5%)	0.525	5 (6.1%)	2 (4.9%)	3 (7.3%)	1.000
Central nervous system	7 (4.1%)	2 (2.2%)	5 (6.5%)	0.156	2 (2.4%)	0 (0.0%)	2 (4.9%)	0.494
Sin and soft tissue	4 (2.4%)	1 (1.1%)	3 (3.9%)	0.330	2 (2.4%)	0 (0.0%)	2 (4.9%)	0.494
*Pathogenic bacteria*								
*DTR-PA*	124 (72.9%)	64 (68.8%)	60 (77.9%)	0.183	53 (64.6%	27 (65.9%)	26 (63.4%)	0.817
Only *CRPA* infection	64 (37.6%)	38 (40.9%)	26 (33.8%)	0.342	30 (36.6%)	12 (29.3%)	18 (43.9%)	0.169
+*CRAB*	59 (34.7%)	37 (39.8%)	22 (28.6%)	0.126	31 (37.8%)	18 (43.9%)	13 (31.7%)	0.255
+*CRKP*	70 (41.2%)	27 (29.0%)	43 (55.8%)	**<0.001**	36 (43.9%)	18 (43.9%)	18 (43.9%)	1.000
+ Other CREs	5 (2.9%)	3 (3.2%)	2 (2.6%)	1.000	3 (3.7%)	2 (4.9%)	1 (2.4%)	1.000
Antibiotic regimens								
Treatment course (day)	10.0 (6.9–14.0)	10.0 (6.8–14.0)	11.0 (6.5–15.0)	0.615	10.0 (7.0–14.0)	8.0 (6.3–12.0)	11.0 (7.5–15.0)	0.057
Combined anti-PA antibiotics	2.0 (1.0–2.0)	2.0 (1.0–2.0)	1.0 (1.0–2.0)	**0.011**	2.0 (1.0–2.0)	2.0 (1.0–2.0)	2.0 (1.0–2.0)	0.947
Monotherapy	67 (39.4%)	28 (30.1%)	39 (50.6%)	**0.006**	39 (47.6%)	19 (46.3%)	20 (48.8%)	0.825
*+ Quinolones*	13 (7.6%)	6 (6.5%)	7 (9.1%)	0.519	6 (7.3%)	2 (4.9%)	4 (9.8%)	0.675
*+ Aminoglycosides*	13 (7.6%)	7 (7.5%)	6 (7.8%)	0.948	8 (9.8%)	5 (12.2%)	3 (7.3%)	0.457
*+Other anti-PA β-lactam*	46 (27.1%)	33 (35.5%)	13 (16.9%)	**0.007**	16 (19.5%)	7 (17.1%)	9 (22.0%)	0.577
*+ Carbapenem*	44 (25.9%)	35 (37.6%)	9 (11.7%)	**<0.001**	19 (23.2%)	12 (29.3%)	7 (17.1%)	0.191
Efficacy and mortality								
Clinical efficacy	87 (51.2%)	45 (48.4%)	42 (54.5%)	0.424	45 (54.9%)	20 (44.4%)	25 (55.6%)	0.267
7-day microbiological clearance	43 (25.3%)	15 (16.1%)	28 (36.4%)	**0.003**	24 (29.3%)	7 (17.1%)	17 (41.5%)	**0.015**
Microbiological clearance	72 (42.4%)	26 (28.0%)	46 (59.7%)	**<0.001**	35 (42.7%)	10 (24.4%)	25 (61.0%)	**0.001**
30-day all-cause mortality	24 (14.1%)	14 (15.1%)	10 (13.0%)	0.700	8 (9.8%)	5 (12.2%)	3 (7.3%)	0.710
AKI	52 (30.6%)	33 (35.5%)	19 (24.7%)	0.128	27 (32.9%)	16 (39.0%)	11 (26.8%)	0.240
Bacterial removal time (days)	8.0 (5.0–13.8)	9.0 (6.5–14.5)	7.0 (4.0–12.5)	0.213	7.0 (5.0–13.8)	9.0 (7.0–14.5)	7.0 (4.0–14.0)	0.254
Survival time (days)	30.0 (3.0–30.0)	30.0 (8.0–30.0)	30.0 (3.0–30.0)	0.492	30.0 (3.0–30.0)	30.0 (8.0–30.0)	30.0 (3.0–30.0)	0.280

Vasoactive drugs include norepinephrine, dopamine, epinephrine, isoproterenol, phentolamine, and nitroglycerin. ICU, Intensive Care Unit. APACHE II, Acute Physiology and Chronic Health Evaluation II; AKI, acute kidney injury; *CRPA*, Carbapenem-resistant *Pseudomonas Aeruginosa*; PMB, polymyxin B; CAZ/AVI, ceftazidime/avibactam; CCR, creatinine clearance rate; CRRT/RRT, continuous renal replacement therapy or intermittent renal replacement therapy; PSM, propensity score-matched. Other anti-PA β-lactam: aztreonam, piperacillin-tazobactam, cefoperazone sulbactam, ceftazidime. Bold font indicates data with significant differences. PSM, variable are bold font indicates data, including vasoactive drugs; ICU, administration, sepsis/septic shock, hypoproteinemia, diabetes mellitus, cardiovascular diseases, multi-site infection, blood infection, abdominal infection, +*CRKP*, combined anti-PA, antibiotics, monotherapy, +other anti-PA β-lactam, + carbapenem. Matching tolerance = 0.2. Randomly arrange the order of cases when selecting matching items. Survival time is expressed by median (minimum-maximum).

**TABLE 2:** Drug sensitivity results of *CRPA*.

Microbial drug sensitivity test	Total (n = 170)	PMB (n = 93)	CAZ/AVI (n = 77)	p-value
Sensitive to PMB (≤2 μg/mL)	170 (100.0%)	93 (100.0%)	77 (100.0%)	1.000
Sensitive to CAZ/AVI (≤8/4 μg/mL)	170 (100.0%)	93 (100.0%)	77 (100.0%)	1.000
Amikacin				
Sensitive (≤16 μg/mL)	70 (41.2%)	32 (34.4%)	38 (49.4%)	0.049
Resistance (>16 μg/mL)	96 (56.5%)	58 (62.4%)	38 (49.4%)	0.088
Ciprofloxacin				
Sensitive (≤0.5 μg/mL)	36 (21.2%)	21 (22.6%)	15 (19.5%)	0.622
Resistance (>0.5 μg/mL)	129 (75.9%)	69 (74.2%)	60 (77.9%)	0.572
Ceftazidime				
Sensitive (≤8 μg/mL)	15 (8.8%)	11 (11.8%)	4 (5.2%)	0.213
Resistance (>8 μg/mL)	146 (85.9%)	74 (79.6%)	72 (93.5%)	0.009

PMB, polymyxin B; CAZ/AVI, ceftazidime/avibactam.

### 3.2 Cohort comparison of PMB and CAZ/AVI in the treatment of *CRPA*

The group that received CAZ/AVI presented with more complex clinical profiles, characterized by a higher burden of comorbidities, diverse infection types, and greater disease severity compared to the PMB-treated cohort. Specifically, CAZ/AVI recipients demonstrated significantly higher rates of vasopressor use (CAZ/AVI:70.1% vs. PMB:53.8%, *p* = 0.029), sepsis/septic shock (CAZ/AVI:48.1% vs. PMB:31.2%, *p* = 0.025), and bloodstream infections (CAZ/AVI:24.7% vs. PMB:6.5%, *p* = 0.001). Additionally, CAZ/AVI monotherapy was more frequently employed than PMB monotherapy (50.6% vs. 30.1%, *p* = 0.006). To address these differences, we performed a 1:1 PSM, resulting in 82 matched cases. Univariate analysis revealed no significant differences in comorbidities, types of infections, or disease severity (details are provided in Table 1).

### 3.3 Treatment outcome

The clinical course analysis included 170 patients undergoing PMB-based regimens or CAZ/AVI-based regimens, with a median treatment duration of 10.0 days (IQR 6.9–14.0). Key therapeutic outcomes demonstrated clinical improvement in 51.2% of cases (87/170), while 14.1% (24/170) either succumbed during hospitalization or required treatment cessation secondary to clinical deterioration. Early microbiological response was observed in 25.3% (43/170) showing *CRPA* clearance within 7 days of antimicrobial initiation. Cumulative *CRPA* clearance rates reached 42.4% (73/170), with a median clearance time of 8.0 days (IQR 5.0–13.8). Treatment-emergent nephrotoxicity manifested as AKI in 30.6% (52/170) at therapy completion.

When comparing antimicrobial regimens, CAZ/AVI-based regimens exhibited superior microbiological efficacy with both 7-day clearance (36.4% vs. 16.1%) and cumulative clearance rates (59.7% vs. 28.0%) demonstrating statistical significance (*p* < 0.05), findings maintained after propensity score adjustment. Nevertheless, intergroup analyses revealed comparable clinical response rates, equivalent 30-day all-cause mortality trajectories, and analogous renal safety profiles (Table 1, “Efficacy and Mortality”). Supplementary Material S2 delineates the dose-response relationships between antimicrobial regimens and treatment outcomes, stratified by dosing intensity.

### 3.4 Microbiological efficacy subgroup analysis

To clarify antibiotic treatment regimens for polymicrobial infections, we conducted detailed medication treatment regimens analyses across three clinical scenarios in Supplementary Material S3. Regarding *CRAB* co-infections (n = 59), these were managed with appropriate adjunct agents (e.g., high-dose sulbactam in 13.5% cases, tigecycline in 13.6%), while CAZ/AVI was specifically used for its anti-*CRPA* activity. Notably, least 23% (n = 15/65) of *CRAB*-positive cases demonstrated persistent colonization patterns (≥3 consecutive positive cultures) without associated inflammatory markers elevation (median CRP 8.2 mg/L vs. 42.7 mg/L in invasive infections, p < 0.001) or organ dysfunction, supporting non-pathogenic carriage status per ESCMID 2023 guidelines. In contrast, *CRPA* detection in these cases was associated with progressive clinical worsening, including rising inflammatory markers and aggravated symptoms.

**FIGURE 2:**
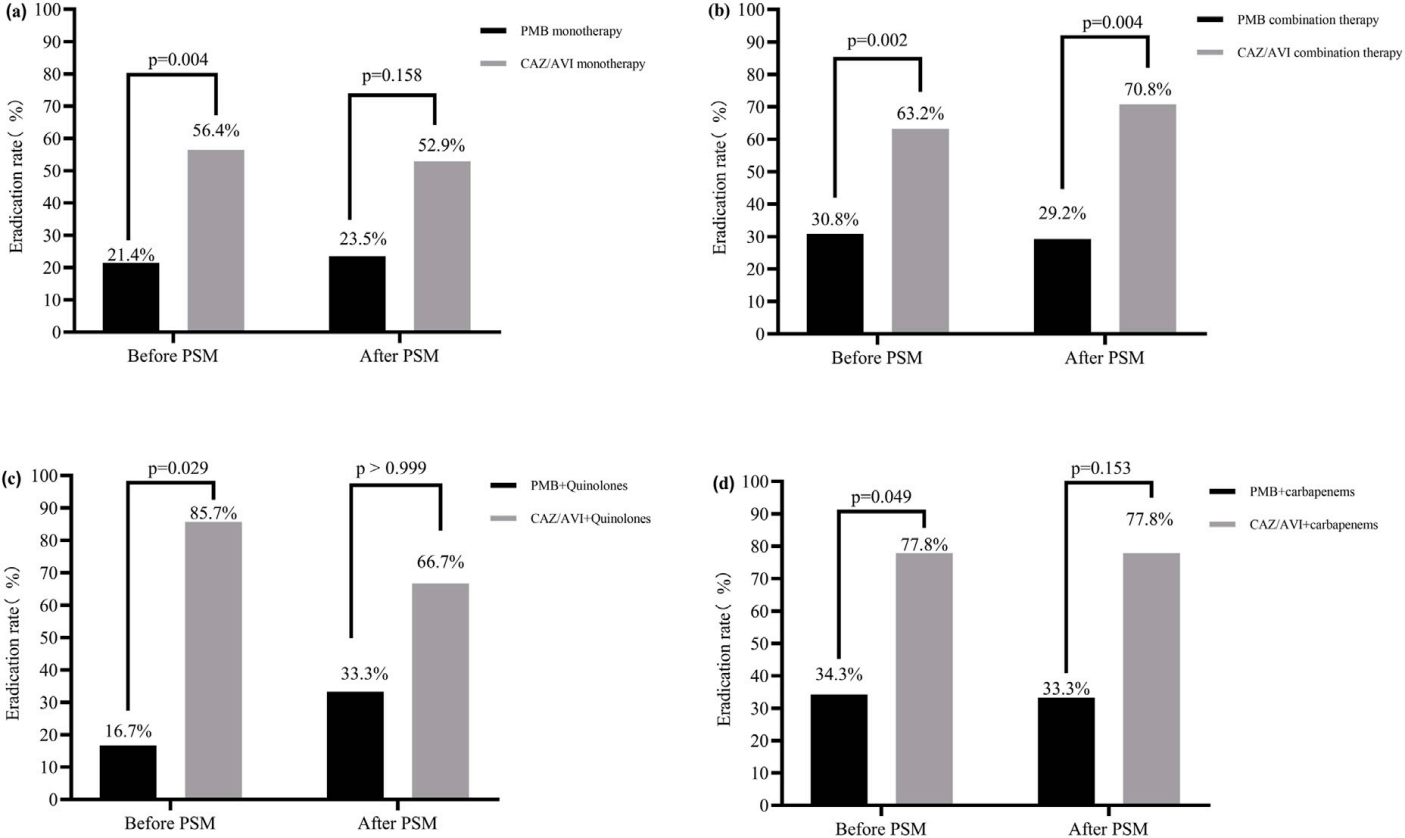
Microbiological efficacy subgroup analysis between PMB and CAZ/AVI-based regimens. **(a)** *CRPA* clearance rate with CAZ/AVI monotherapy vs PMB monotherapy; **(b)** *CRPA* clearance rate with CAZ/AVI combination therapy vs PMB combination therapy; **(c)** *CRPA* clearance rate with CAZ/AVI combined with quinolones vs. PMB combined with quinolones; **(d)** *CRPA* clearance rate with CAZ/AVI combined with carbapenems vs PMB combined with carbapenems.

Although there was no significant difference in the clearance rate of *CRPA* between monotherapy and combination therapy (*p* > 0.05), Subgroup analysis revealed that, after PSM, the CAZ/AVI-based combination regimens achieved significantly higher CRPA clearance rates compared to the PMB-based combination regimens (70.8% vs. 29.2%, *p* = 0.004) (Figure 2b). However, no statistically significant differences in CRPA clearance were observed in the monotherapy subgroup or other combination therapy subgroups (*p* > 0.05) (Figures 2a, c, d). Detailed results are presented in Figure 2; Supplementary Materials S4, S5.

### 3.5 Factors influencing clinical efficacy

To better understand the clinical outcomes of *CRPA* infections, we conducted a further analysis of factors influencing the clinical efficacy of CAZ/AVI and PMB in treating *CRPA* infections. In the cohort study comparing the success and failure groups, data both before and after PSM revealed that patients in the failure group had a higher severity of illness, as evidenced by a greater need for Mechanical ventilation (88.0% vs. 67.8%, *p* = 0.002) and vasoactive agent use (74.7% vs. 48.3%, *p* < 0.001), and the infection situation were more complex, as *DTR-PA* infections (81.9% vs. 64.4%, *p* = 0.010), compared to the success group (Table 3). Multivariate analysis (Table 4) identified the following influencing factors before PSM: CAZ/AVI-based regimens (OR 0.596,95%CI 0.305–1.166, *p* = 0.131), *DTR-PA* infections (OR 2.272, 95%CI 1.060–4.869, *p* = 0.035), and the use of vasoactive agents (OR 2.399, 95%CI 1.137–5.161, *p* = 0.022). However, PSM-adjusted multivariable analysis revealed no clinically significant differences in baseline characteristics (*p* > 0.05).

**TABLE 3:** Univariate analysis of factors associated with clinical efficacy in *CRPA*-infected patients.

Demographics and clinical characteristics	Before PSM			After PSM		
	Success (N = 87)	Failure (N = 83)	P-value	Success (N = 45)	Failure (N = 37)	P-value
Demographic characteristics						
Age (years)	60.2 ± 17.5	62.1 ± 17.8	0.472	59.3 ± 16.9	63.2 ± 19.8	0.175
Gender (male)	69 (79.3%)	68 (81.9%)	0.666	34 (75.6%)	32 (86.5%)	0.214
Baseline creatinine (umol/L)	67.1 (47.4–136.0)	81.9 (54.2–117.0)	0.274	67.1 (45.0–173.3)	88.8 (58.7–117.0)	0.447
Baseline CCR (mL/min)	81.0 (37.2–121.1)	71.1 (40.5–90.6)	0.180	84.0 (29.5–113.4)	64.6 (42.6–83.2)	0.378
CRRT/RRT	14 (16.1%)	10 (12.0%)	0.449	10 (22.2%)	4 (10.8%)	0.172
Mechanical ventilation	59 (67.8%)	73 (88.0%)	**0.002**	29 (64.4%)	31 (51.7%)	**0.049**
Vasoactive drugs	42 (48.3%)	62 (74.7%)	**<0.001**	21 (46.7%)	26 (70.3%)	**0.032**
ICU administration	65 (74.7%)	58 (69.9%)	0.481	33 (73.3%)	21 (56.8%)	0.115
Sepsis/septic shock	33 (37.9%)	33 (39.8%)	0.807	20 (44.4%)	16 (43.2%)	0.913
Hospital stays (days)	42.0 (25.0–63.0)	37.0 (22.0–55.0)	0.240	42.0 (24.0–65.0)	33.0 (19.5–48.5)	0.158
APACHE II score	23.0 (20.0–23.0)	23.0 (19.0–28.0)	0.142	22.0 (19.0–23.0)	23.0 (19.5–28.0)	0.183
Comorbidity						
Solid organ transplantation	2 (2.3%)	4 (4.8%)	0.435	0 (0.0%)	2 (5.4%)	0.201
Hypoproteinemia	31 (35.6%)	21 (25.3%)	0.144	15 (33.3%)	10 (27.0%)	0.537
Respiratory diseases	74 (85.1%)	74 (89.2%)	0.426	36 (80.0%)	32 (86.5%)	0.437
Renal insufficiency	17 (19.5%)	9 (10.8%)	0.115	7 (15.6%)	5 (13.5%)	0.795
Diabetes mellitus	24 (27.6%)	18 (21.7%)	0.373	11 (24.4%)	11 (29.7%)	0.591
Urinary system disease	15 (17.2%)	11 (13.3%)	0.470	8 (17.8%)	4 (10.8%)	0.374
Digestive system diseases	29 (33.3%)	32 (38.6%)	0.478	18 (40.0%)	16 (43.2%)	0.767
Abnormal liver function	17 (19.5%)	19 (22.9%)	0.593	9 (20.0%)	11 (29.7%)	0.307
Cerebrovascular diseases	52 (59.8%)	38 (45.8%)	0.068	20 (44.4%)	19 (51.4%)	0.533
Cardiovascular diseases	53 (60.9%)	44 (53.0%)	0.298	26 (57.8%)	22 (59.5%)	0.878
Malignancy	12 (13.8%)	14 (16.9%)	0.578	5 (11.1%)	7 (18.9%)	0.320
*Infection sites*						
Multi-site infection	21 (24.1%)	23 (27.7%)	0.595	14 (31.1%)	11 (29.7%)	0.892
Respiratory tract	81 (93.1%)	74 (89.2%)	0.364	43 (95.6%)	33 (89.2%)	0.402
Blood	10 (11.5%)	15 (18.1%)	0.226	6 (13.3%)	8 (21.6%)	0.321
Abdominal	7 (8.0%)	10 (12.0%)	0.385	6 (13.3%)	6 (16.2%)	0.713
Urinary tract	4 (4.6%)	5 (6.0%)	0.942	2 (4.4%)	3 (8.1%)	0.654
Central nervous system	4 (4.6%)	3 (3.6%)	1.000	1 (2.2%)	1 (2.7%)	1.000
Sin and soft tissue	3 (3.4%)	1 (1.2%)	0.621	2 (4.4%)	0 (0.0%)	0.499
*Pathogenic bacteria*						
*DTR-PA*	56 (64.4%)	68 (81.9%)	**0.010**	25 (55.6%)	28 (75.7%)	0.058
Only *CRPA* infection	36 (41.4%)	28 (33.7%)	0.304	27 (60.0%)	25 (67.6%)	0.479
*+CRAB*	27 (31.0%)	32 (38.6%)	0.303	17 (37.8%)	14 (37.8%)	0.996
*+CRKP*	37 (42.5%)	33 (39.8%)	0.714	20 (44.4%)	16 (43.2%)	0.913
*+ Other CREs*	0 (0.0%)	5 (6.0%)	0.062	0 (0.0%)	3 (8.1%)	0.088
Antibiotic regimens						
Treatment course (day)	11.0 (7.0–15.0)	8.5 (6.0–14.0)	0.064	11.0 (7.0–14.0)	8.0 (5.5–12.0)	**0.032**
Combined antibiotics of *anti-PA*	2.0 (1.0–2.0)	2.0 (1.0–2.0)	0.196	2.0 (1.0–2.0)	1.0 (1.0–2.0)	0.235
Monotherapy	31 (35.6%)	36 (43.4%)	0.302	19 (42.2%)	20 (54.1%)	0.286
*+ Quinolones*	5 (5.7%)	8 (9.6%)	0.340	4 (8.9%)	2 (5.4%)	0.547
*+ Aminoglycosides*	7 (8.0%)	6 (7.2%)	0.841	3 (6.7%)	5 (13.5%)	0.506
*+Other β-lactam of anti-PA*	26 (29.9%)	20 (24.1%)	0.396	11 (24.4%)	5 (13.5%)	0.214
*+ Carbapenem*	28 (32.2%)	16 (19.3%)	0.055	12 (26.7%)	7 (18.9%)	0.408
CAZ/AVI-based regimens	42 (48.3%)	35 (42.2%)	0.424	25 (55.6%)	16 (43.2%)	0.267

Abbreviations are the same as Table 1. Bold font indicates data with significant differences.

**TABLE 4:** Binary logistic regressive analysis of factors associated with clinical efficacy.

Demographics and clinical characteristics	Before PSM			After PSM		
	B	Or (95% CI)	P-value	B	Or (95% CI)	P-value
CAZ/AVI-based regimens	−0.517	0.596 (0.305–1.166)	**0.131**	−0.492	0.611 (0.229–1.632)	0.326
DTR-PA	0.821	2.272 (1.060–4.869)	**0.035**	0.801	2.227 (0.807–6.146)	0.122
Mechanical ventilation	0.728	2.071 (0.851–5.143)	0.109	0.514	1.671 (0.482–5.800)	0.419
Vasoactive drugs	0.875	2.399 (1.137–5.161)	**0.022**	0.816	2.262 (0.762–6.713)	0.141
Treatment course (day)	−0.026	0.97590.930–1.022)	0.284	−0.069	0.933 (0.847–1.0280	0.161

The multivariate analysis model included all variables with p < 0.05 from the univariate analysis of data before and after PSM, as well as variables fixed based on CAZ/AVI and DTR-PA. Bold font indicates data with significant differences.

### 3.6 Factors influencing microbiological clearance

**FIGURE 3:**
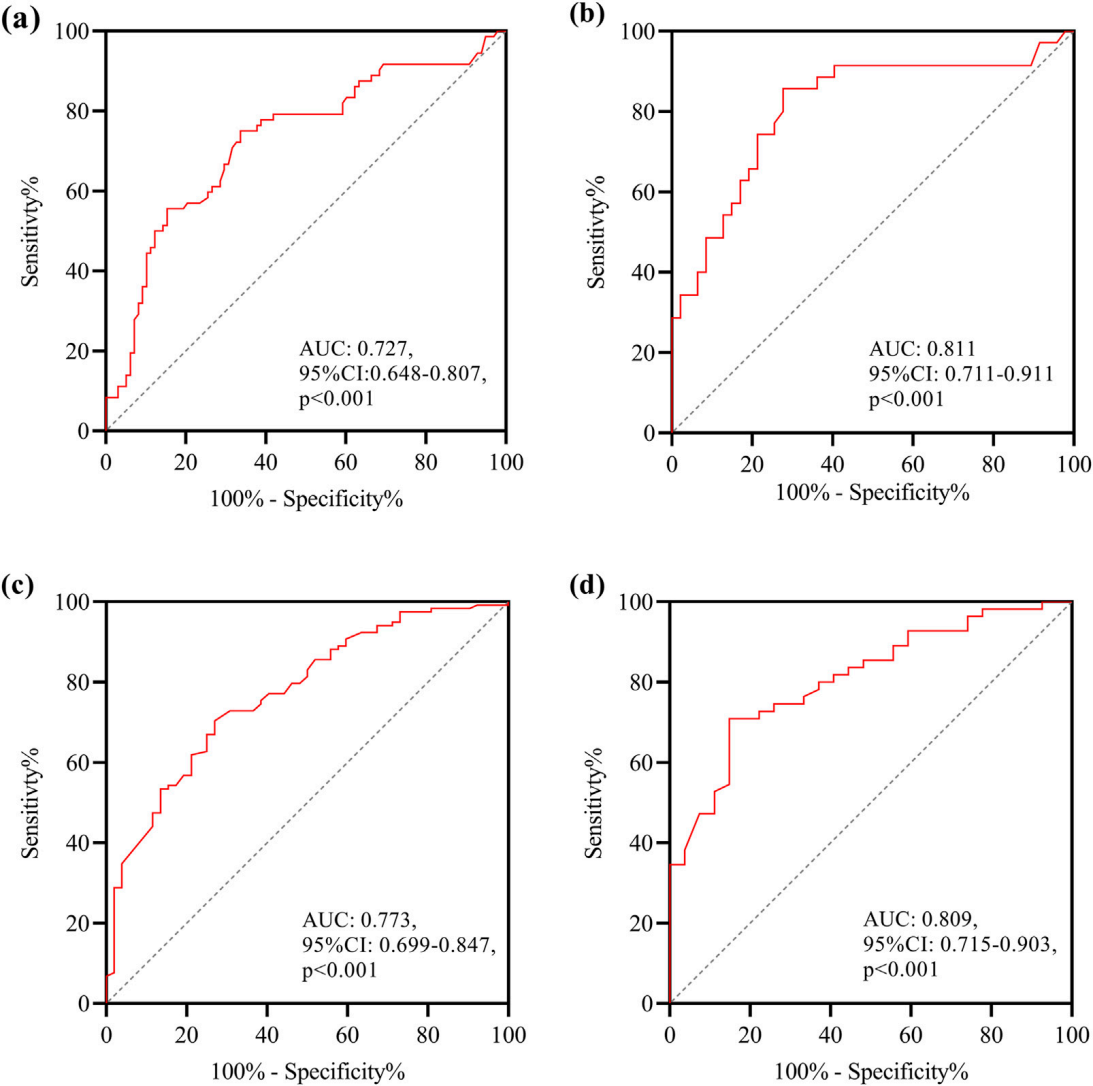
The multifactor analysis model ROC curve for predicting the occurrence of microbiological clearance and AKI. **(a)** Data before PSM for predicting the occurrence of microbiological clearance failure; **(b)** data after PSM for predicting the occurrence of microbiological clearance failure; **(c)** data before PSM for predicting the occurrence of AKI; **(d)** data after PSM for predicting the occurrence of AKI.

In the analysis of factors influencing the clearance rate of *CRPA* infections treated with PMB and CAZ/AVI, a comparison between the clearance success group and the clearance failure group (Table 5) revealed the following findings based on data before and after PSM. Regarding the median differences, the duration of treatment in the clearance failure group was significantly shorter than that in the clearance success group [9.0 (5.9–14.0) vs. 11.0 (8.0–15.0), *p* = 0.038]. In terms of treatment regimens, the utilization rate of CAZ/AVI in the clearance failure group was lower than that in the clearance success group (31.6% vs. 63.9%, *p* < 0.001). Multivariate analysis identified the following influencing factors before PSM: treatment with CAZ/AVI (OR 0.218, 95%CI 0.108–0.440, *p* < 0.001) and *DTR-PA* infections (OR 2.139, 95%CI 1.011–4.529, *p* = 0.047). After PSM, treatment with CAZ/AVI remained a significant protective factor (OR 0.185, 95%CI 0.061–0.564, *p* = 0.003). Furthermore, multi-site infection (OR 0.295, 95%CI 0.097–0.899, *p* = 0.032) and the number of combined anti-PA antibiotics (OR 0.435, 95%CI 0.213–0.888, *p* = 0.022) were identified as protective factors associated with improved CRPA clearance rates (Table 6). The ROC curves demonstrated robust discriminatory performance of the multivariable regression model, with AUC values maintaining >0.70 across sensitivity analyses (Figures 3a, b).

**TABLE 5:** Univariate analysis of factors associated with microbiological efficacy in *CRPA*-infected patients.

Demographics and clinical characteristics	Before PSM			After PSM		
	CRPA clearance success (N = 72)	CRPA clearance failure (N = 98)	P-value	CRPA clearance success (N = 35)	CRPA clearance failure (N = 47)	P-value
Demographic characteristics						
Age (years)	58.3 ± 18.8	63.3 ± 16.4	0.067	57.6 ± 19.3	63.7 ± 17.2	0.136
Gender (male)	58 (80.6%)	79 (80.6%)	0.993	29 (82.9%)	37 (78.7%)	0.640
Baseline creatinine (umol/L)	72.4 (49.0–159.0)	78.3 (49.2–117.0)	0.830	87.6 (53.3–195.1)	79.0 (48.9–117.0)	0.511
Baseline CCR (mL/min)	73.5 (34.3–120.9)	73.8 (40.3–104.8)	0.940	73.6 (29.0–102.3)	75.6 (40.2–104.5)	0.757
CRRT/RRT	13 (18.1%)	11 (11.2%)	0.206	8 (22.9%)	6 (12.8%)	0.230
Mechanical ventilation	55 (76.4%)	77 (78.6%)	0.736	24 (68.6%)	36 (76.6%)	0.417
Vasoactive drugs	49 (68.1%)	55 (56.1%)	0.115	23 (65.7%)	24 (51.1%)	0.185
ICU administration	50 (69.4%)	73 (74.5%)	0.467	23 (65.7%)	31 (66.0%)	0.982
Sepsis/septic shock	32 (44.4%)	34 (51.5%)	0.197	17 (48.6%)	19 (40.4%)	0.462
Hospital stays (days)	38.0 (24.3–50.8)	39.5 (24.8–67.5)	0.401	38.0 (24.0–49.0)	39.0 (22.0–72.0)	0.732
APACHE II score	23.0 (21.0–26.0)	23.0 (19.0–25.0)	0.194	23.0 (19.0–26.0)	22.0 (19.0–23.0)	0.269
Comorbidity						
Solid organ transplantation	3 (4.2%)	3 (3.1%)	0.699	0 (0.0%)	2 (4.3%)	0.505
Hypoproteinemia	26 (36.1%)	26 (26.5%)	0.180	12 (34.3%)	13 (27.7%)	0.519
Respiratory diseases	63 (87.5%)	85 (86.7%)	0.883	31 (88.6%)	37 (78.7%)	0.381
Renal insufficiency	11 (15.3%)	15 (15.3%)	0.996	5 (14.3%)	7 (14.9%)	0.939
Diabetes mellitus	18 (25.0%)	24 (24.5%)	0.939	9 (25.7%)	13 (27.7%)	0.844
Urinary system disease	12 (16.7%)	14 (14.3%)	0.670	8 (22.9%)	4 (8.5%)	0.069
Digestive system diseases	30 (41.7%)	31 (31.6%)	0.178	18 (51.4%)	16 (34.0%)	0.114
Abnormal liver function	19 (26.4%)	17 (17.3%)	0.154	10 (28.6%)	10 (21.3%)	0.447
Cerebrovascular diseases	37 (51.4%)	53 (54.1%)	0.728	17 (48.6%)	22 (46.8%)	0.874
Cardiovascular diseases	43 (59.7%)	54 (55.1%)	0.548	21 (60.0%)	27 (57.4%)	0.816
Malignancy	15 (20.8%)	11 (11.2%)	0.085	8 (22.9%)	4 (8.5%)	0.133
*Infection sites*						
Multi-site infection	24 (33.3%)	20 (20.4%)	0.057	16 (45.7%)	9 (19.1%)	**0.010**
Respiratory tract	64 (88.9%)	91 (92.9%)	0.367	32 (91.4%)	44 (93.6%)	1.000
Blood	14 (19.4%)	11 (11.2%)	0.135	9 (25.7%)	5 (10.6%)	0.073
Abdominal	9 (12.5%)	8 (8.2%)	0.352	8 (22.9%)	4 (8.5%)	0.133
Urinary tract	4 (5.6%)	5 (5.1%)	0.896	3 (8.6%)	2 (4.3%)	0.646
Central nervous system	5 (6.9%)	2 (2.0%)	0.230	1 (2.9%)	1 (2.1%)	1.000
Sin and soft tissue	1 (1.4%)	3 (3.1%)	0.638	1 (2.9%)	1 (2.1%)	1.000
*Pathogenic bacteria*						
*DTR-PA*	48 (66.7%)	76 (71.5%)	0.114	19 (54.3%)	34 (72.3%)	0.091
Only *CRPA* infection	40 (55.6%)	66 (67.3%)	0.117	15 (42.9%)	15 (31.9%)	0.309
+CRAB	22 (30.6%)	37 (37.8%)	0.330	12 (34.3%)	19 (40.4%)	0.571
+*CRKP*	26 (36.1%)	44 (44.9%)	0.250	12 (34.3%)	24 (51.1%)	0.130
+ Other CREs	1 (1.4%)	4 (4.1%)	0.397	1 (2.9%)	2 (4.3%)	0.739
Antibiotic regimens						
Treatment course (days)	11.0 (8.0–15.0)	9.0 (5.9–14.0)	**0.038**	11.0 (8.0–15.0)	8.0 (5.0–13.0)	**0.030**
Combined antibiotics of *anti-PA*	2.0 (1.0–2.0)	2.0 (1.0–2.0)	0.451	2.0 (1.0–3.0)	1.0 (1.0–2.0)	**0.029**
Monotherapy	28 (38.9%)	39 (39.8%)	0.905	13 (37.1%)	26 (55.3%)	0.103
*+ Quinolones*	7 (9.7%)	6 (6.1%)	0.383	4 (11.4%)	2 (4.3%)	0.394
*+ Aminoglycosides*	3 (4.2%)	10 (10.2%)	0.241	2 (5.7%)	6 (12.8%)	0.491
*+Other β-lactam of anti-PA*	21 (29.2%)	25 (25.5%)	0.596	10 (28.6%)	6 (12.8%)	0.074
*+ Carbapenem*	19 (26.4%)	25 (25.5%)	0.897	10 (28.6%)	9 (19.1%)	0.317
CAZ/AVI-based regimens	46 (63.9%)	31 (31.6%)	**<0.001**	25 (71.4%)	16 (34.0%)	**0.001**

Abbreviations are the same as Table 1. Bold font indicates data with significant differences.

**TABLE 6:** Binary logistic regressive analysis of factors associated with microbiological efficacy.

Demographics and clinical characteristics	Before PSM			After PSM		
	B	Or (95% CI)	P-value	B	Or (95% CI)	P-value
CAZ/AVI-based regimens	−1.525	0.218 (0.108–0.440)	**<0.001**	−1.687	0.185 (0.061–0.564)	**0.003**
*DTR-PA*	0.761	2.139 (1.011–4.529)	**0.047**	0.814	2.256 (0.770–6.617)	0.138
Multi-site infection	−0.378	0.686 (0.324–1.450)	0.323	−1.221	0.295 (0.097–0.899)	**0.032**
Treatment course (days)	−0.026	0.974 (0.933–1.016)	0.224	−0.009	0.991 (0.905–1.085)	0.842
Combined antibiotics of *anti-PA*	−0.314	0.730 (0.478–1.117)	0.147	−0.833	0.435 (0.213–0.888)	**0.022**

The multivariate analysis model included all variables with p < 0.05 from the univariate analysis of data before and after PSM, as well as variables fixed based on CAZ/AVI and DTR-PA. Bold font indicates data with significant differences.

### 3.7 Factors influencing acute kidney injury

To evaluate the safety of PMB and CAZ/AVI in the treatment of *CRPA* infections, we conducted an analysis of factors influencing the development of AKI. In the comparison between the AKI group and the non-AKI group (Table 7), the AKI group exhibited higher rates of comorbidities, disease severity, and specific infection types compared to the non-AKI group. However, there were no significant differences between the AKI group and the non-AKI group in terms of baseline creatinine values [101.6 (54.7–160.7) vs. 71.4 (49.0–117.0), *p* = 0.098], creatinine clearance rates [66.9 (36.7–89.4) vs. 79.0 (40.3–118.6), *p* = 0.085], or the use of combined antibiotics of anti-PA [2.0 (1.0–2.0) vs. 2.0 (1.0–2.0), *p* = 0.880]. Multivariate analysis (Table 8) identified the following influencing factors before PSM: hypoproteinemia (OR 0.375, 95%CI 0.146–0.9620, *p* = 0.041), renal insufficiency (OR 5.360, 95% CI 1.929–14.898, *p* = 0.001), diabetes mellitus (OR 2.778, 95% CI 1.166–6.623, *p* = 0.027), digestive system diseases (OR 2.503, 95% CI 1.094–5.726, *p* = 0.030), and PMB-based regimens (OR 2.510, 95%CI 1.053–5.984, *p* = 0.038). After PSM, diabetes mellitus remained a significant influencing factor (OR 3.600, 95%CI 1.018–12.733, *p* = 0.047), and the sepsis/septic shock (OR 3.405, 95%CI 1.007–11.520, *p* = 0.049) increased the risk of AKI. The ROC curves demonstrated robust discriminatory performance of the multivariable regression model, with AUC values maintaining >0.70 across sensitivity analyses (Figures 3c, d).

**TABLE 7:** Univariate analysis of factors associated with AKI in *CRPA*-infected patients.

Demographics and clinical characteristics	Before PSM			After PSM		
	Non-AKI (N = 118)	AKI (N = 52)	P-value	Non-AKI (N = 55)	AKI (N = 27)	P-value
Demographic characteristics						
Age (years)	60.2 ± 18.2	63.3 ± 16.2	0.302	60.4 ± 18.7	62.4 ± 17.6	0.652
Gender (male)	93 (78.8%)	44 (84.6%)	0.379	45 (81.8%)	21 (77.8%)	0.664
Baseline creatinine (umol/L)	71.4 (49.0–117.0)	101.6 (54.7–160.7)	0.098	78.0 (49.2–125.0)	115.8 (46.9–149.9)	0.366
Baseline CCR (mL/min)	79.0 (40.3–118.6)	66.9 (36.7–89.4)	0.085	75.6 (38.8–111.0)	73.3 (22.2–95.8)	0.351
RRT	14 (11.9%)	10 (19.2%)	0.204	8 (14.5%)	6 (22.2%)	0.385
Mechanical ventilation	88 (74.6%)	44 (84.6%)	0.148	37 (67.3%)	23 (85.2%)	0.146
Vasoactive drugs	71 (60.2%)	33 (63.5%)	0.685	27 (49.1%)	20 (74.1%)	**0.032**
ICU administration	85 (72.0%)	38 (73.1%)	0.889	35 (63.6%)	19 (70.4%)	0.546
Sepsis/septic shock	39 (33.1%)	27 (51.9%)	**0.020**	19 (34.5%)	17 (63.0%)	**0.015**
Hospital stays (days)	38.0 (22.0–55.3)	39.5 (27.5–66.5)	0.255	39.0 (21.0–58.0)	30.0 (24.0–55.0)	0.564
APACHE II score	23.0 (21.0–26.0)	23.0 (18.0–25.0)	0.139	23.0 (21.0–23.0)	20.0 (12.0–28.0)	0.337
Comorbidity						
Solid organ transplantation	2 (1.7%)	4 (7.7%)	0.072	0 (0.0%)	2 (7.4%)	0.106
Hypoproteinemia	42 (35.6%)	10 (19.2%)	**0.033**	18 (32.7%)	7 (25.9%)	0.530
Respiratory diseases	104 (88.1%)	44 (84.6%)	0.529	45 (81.8%)	23 (85.2%)	0.945
Renal insufficiency	11 (9.3%)	15 (28.8%)	**0.001**	6 (10.9%)	6 (22.2%)	0.173
Diabetes mellitus	24 (20.3%)	18 (34.6%)	**0.047**	12 (21.8%)	10 (37.0%)	0.144
Urinary system disease	15 (12.7%)	11 (21.2%)	0.159	6 (10.9%)	6 (22.2%)	0.173
Digestive system diseases	36 (30.5%)	25 (48.1%)	**0.028**	16 (29.1%)	14 (51.9%)	**0.044**
Abnormal liver function	26 (22.0%)	10 (19.2%)	0.680	12 (21.8%)	8 (29.6%)	0.439
Cerebrovascular diseases	67 (56.8%)	23 (44.2%)	0.131	28 (50.9%)	11 (40.7%)	0.386
Cardiovascular diseases	66 (55.9%)	31 (59.6%)	0.655	30 (54.5%)	18 (66.7%)	0.295
Malignancy	18 (15.3%)	8 (15.4%)	0.983	9 (16.4%)	3 (11.1%)	0.764
*Infection sites*						
Multi-site infection	27 (22.9%)	17 (32.7%)	0.178	17 (30.9%)	8 (29.6%)	0.906
Respiratory tract	107 (90.7%)	48 (92.3%)	0.959	52 (94.5%)	24 (88.9%)	0.390
Blood	13 (11.0%)	12 (23.1%)	**0.041**	8 (14.5%)	6 (22.2%)	0.385
Abdominal	9 (7.6%)	8 (15.4%)	0.120	5 (9.1%)	7 (25.9%)	**0.043**
Urinary tract	7 (5.9%)	2 (3.8%)	0.851	4 (7.3%)	1 (3.7%)	>0.999
Central nervous system	7 (5.9%)	0 (0.0%)	0.169	2 (3.6%)	0 (0.0%)	1.000
Sin and soft tissue	4 (3.4%)	0 (0.0%)	0.314	2 (3.6%)	0 (0.0%)	1.000
*Pathogenic bacteria*						
*DTR-PA*	89 (75.4%)	35 (67.3%)	0.272	33 (60.0%)	20 (74.1%)	0.210
Only *CRPA* infection	47 (39.8%)	17 (32.7%)	0.376	24 (43.6%)	6 (22.2%)	0.058
+*CRAB*	42 (35.6%)	17 (32.7%)	0.714	21 (38.2%)	10 (37.0%)	0.920
+*CRKP*	46 (39.0%)	24 (46.2%)	0.381	22 (40.0%)	14 (51.9%)	0.309
+ Other *CREs*	3 (2.5%)	2 (3.8%)	0.642	1 (1.8%)	2 (7.4%)	0.251
Antibiotic regimens						
Treatment course (day)	11.0 (7.0–15.0)	9.0 (6.0–13.8)	0.181	10.0 (7.0–14.0)	9.0 (7.0–12.0)	0.363
Combined antibiotics of *anti-PA*	2.0 (1.0–2.0)	2.0 (1.0–2.0)	0.880	2.0 (1.0–2.0)	2.0 (1.0–2.0)	0.996
Monotherapy	48 (40.7%)	19 (36.5%)	0.611	26 (47.3%)	13 (48.1%)	0.941
*+ Quinolones*	11 (9.3%)	2 (3.8%)	0.355	5 (9.1%)	1 (3.7%)	0.668
*+ Aminoglycosides*	11 (9.3%)	2 (3.8%)	0.355	7 (12.7%)	1 (0.7%)	0.369
*+Other β-lactam of anti-PA*	28 (23.7%)	18 (34.6%)	0.141	11 (20.0%)	5 (18.5%)	0.874
*+ Carbapenem*	32 (27.1%)	12 (23.1%)	0.579	12 (21.8%)	7 (25.9%)	0.679
PMB-based regimens	60 (50.8%)	33 (63.5%)	0.128	25 (45.5%)	16 (59.3%)	0.240

Abbreviations are the same as Table 1. Bold font indicates data with significant differences.

**TABLE 8:** Binary logistic regressive analysis of factors associated with AKI.

Demographics and clinical characteristics	Before PSM			After PSM		
	B	Or (95% CI)	P-value	B	Or (95% CI)	P-value
PMB-based regimens	0.920	2.510 (1.053–5.984)	**0.038**	0.771	2.161 (0.686–6.813)	0.188
*DTR-PA*	−0.399	0.671 (0.286–1.577)	0.360	0.954	2.596 (0.737–9.149)	0.138
Sepsis/septic shock	0.637	1.891 (0.814–4.395)	0.139	1.225	3.405 (1.007–11.520)	**0.049**
Vasoactive drugs	0.099	1.105 (0.492–2.481)	0.810	0.740	2.096 (0.664–6.619)	0.207
Hypoproteinemia	−0.981	0.375 (0.146–0.962)	**0.041**	−0.567	0.567 (0.148–2.173)	0.408
Renal insufficiency	1.679	5.360 (1.929–14.898)	**0.001**	0.779	2.179 (0.457–10.377)	0.328
Diabetes mellitus	1.022	2.778 (1.166–6.623)	**0.021**	1.281	3.600 (1.018–12.733)	**0.047**
Digestive system diseases	0.917	2.503 (1.094–5.726)	**0.030**	0.949	2.583 (0.667–9.999)	0.169
Blood infection	0.145	1.155 (0.385–3.469)	0.797	−0.871	0.419 (0.084–2.080)	0.287
Abdominal infection	−0.188	0.828 (0.240–2.865)	0.766	0.292	1.338 (0.254–7.054)	0.731

The multivariate analysis model included all variables with p < 0.05 from the univariate analysis of data before and after PSM, as well as variables fixed based on CAZ/AVI and DTR-PA. Bold font indicates data with significant differences.

### 3.8 Factors influencing 30-day all-cause mortality

The all-cause mortality rates at 30 days after treatment with PMB-based regimens and CAZ/AVI-based regimens were 15.1% (14/93) and 13.0% (10/77), respectively. Incorporating microbiological efficacy outcomes and the incidence of AKI into the analysis of 30-day all-cause mortality (Table 9), the univariate analysis showed that the proportion of patients with sepsis-induced shock in the non-survival group was significantly higher than that in the survival group before and after PSM (before PSM: 66.6% vs. 34.2%, p = 0.003; after PSM: 87.5% vs. 39.1%, *p* = 0.025). The median APACHE II score was also higher in the non-survival group compared to the survival group [before PSM: 29.00 (23.00–36.25) vs. 23.00 (19.00–23.00), *p* < 0.001], but the difference in APACHE II scores between the two groups was not statistically significant after PSM. The multivariate COX regression analysis revealed that sepsis/septic shock (HR 2.702, 95%CI 1.115–6.548, *p* = 0.028), APACHE II (HR 1.072, 95%CI 1.032–1.114, *p* < 0.001) were independent risk factors for 30-day all-cause mortality in patients with *CRPA* infection, with APACHE II (HR 1.103, 95%CI 1.105–1.198, *p* = 0.021) remained statistically significant in the multivariate analysis after PSM, and the other *CREs* infections (HR 40.849, 95%CI 3.323–502.170, *p* = 0.004) increased the risk of 30-day all-cause mortality (Table 10). Notably, neither *DTR-PA* infection status (HR 0.916, 95%CI 0.340–2.471) nor the treatment selection between CAZ/AVI and PMB regimens (HR 2.426, 95%CI 0.886–6.646) showed significant impact on 30-day all-cause mortality outcomes in the multivariate COX regression analysis (*p* > 0.05).

**TABLE 9:** Univariate analysis of factors associated with 30-day all-cause mortality in *CRPA*-infected patients.

Demographics and clinical characteristics	Before PSM			After PSM		
	Survival (N = 146)	Non-survival (N = 24)	P-value	Survival (N = 78)	Non-survival (N = 8)	P-value
Demographic characteristics						
Age (years)	60.35 ± 17.85	66.00 ± 15.49	0.145	60.28 ± 18.47	68.25 ± 15.24	0.243
Gender (male)	119 (81.5%)	18 (75.0%)	0.639	60 (81.0%)	6 (75.0%)	>0.999
Baseline creatinine (umol/L)	77.00 (49.00–117.00)	74.95 (54.25–158.18)	0.847	82.25 (48.92–155.25)	84.00 (66.92–119.00)	0.684
Baseline CCR (mL/min)	73.56 (40.60–108.42)	69.64 (38.63–101.82)	0.714	73.43 (37.04–104.91)	82.71 (52.15–88.57)	0.988
RRT	19 (13.0%)	5 (20.8%)	0.482	12 (16.2%)	2 (25.0%)	0.894
Mechanical ventilation	110 (75.3%)	22 (91.6%)	0.075	53 (71.6%)	7 (87.5%)	0.587
Vasoactive drugs	83 (56.8%)	21 (87.5%)	**0.004**	41 (55.4%)	6 (75.0%)	0.491
ICU administration	104 (71.2%)	19 (79.1%)	0.421	48 (64.8%)	6 (75.0%)	0.856
Sepsis/septic shock	50 (34.2%)	16 (66.6%)	**0.003**	29 (39.1%)	7 (87.5%)	**0.025**
Hospital stays (days)	40.00 (25.00–62.75)	34.50 (23.75–45.50)	0.235	38.50 (22.00–61.00)	33.00 (24.00–38.25)	0.462
APACHE II score	23.00 (19.00–23.00)	29.00 (23.00–36.25)	**<0.001**	23.00 (19.00–23.00)	26.00 (17.75–39.50)	0.309
Comorbidity						
Solid organ transplantation	4 (2.7%)	2 (8.3%)	0.201	1 (1.3%)	1 (12.5%)	0.187
Hypoproteinemia	44 (30.1%)	8 (33.3%)	0.753	21 (28.3%)	4 (50.0%)	0.391
Respiratory diseases	126 (86.3%)	22 (91.6%)	0.691	60 (81.0%)	8 (100.0%)	0.392
Renal insufficiency	25 (17.1%)	1 (4.1%)	0.184	12 (16.2%)	0 (0.0%)	0.480
Diabetes mellitus	34 (23.2%)	8 (33.3%)	0.290	19 (25.6%)	3 (37.5%)	0.766
Urinary system disease	22 (15.0%)	4 (16.6%)	>0.999	11 (14.8%)	1 (12.5%)	>0.999
Digestive system diseases	49 (33.5%)	12 (50.0%)	0.120	26 (35.1%)	4 (50.0%)	0.658
Abnormal liver function	29 (19.8%)	7 (29.1%)	0.301	18 (24.3%)	2 (25.0%)	>0.999
Cerebrovascular diseases	80 (54.7%)	10 (41.6%)	0.232	36 (48.6%)	3 (37.5%)	0.820
Cardiovascular diseases	82 (56.1%)	15 (62.5%)	0.561	41 (55.4%)	7 (87.5%)	0.170
Malignancy	23 (15.7%)	3 (12.5%)	0.917	10 (13.5%)	2 (25.0%)	0.729
*Infection sites*						
Multi-site infection	36 (24.6%)	8 (33.3%)	0.368	21 (28.3%)	4 (50.0%)	0.391
Respiratory tract	130 (89.0%)	20 (83.3%)	0.644	66 (89.1%)	7 (87.5%)	>0.999
Blood	19 (13.0%)	7 (29.1%)	0.083	10 (13.5%)	3 (37.5%)	0.209
Abdominal	16 (10.9%)	2 (8.3%)	0.976	12 (16.2%)	1 (12.5%)	>0.999
Urinary tract	9 (6.1%)	2 (8.3%)	>0.999	6 (8.1%)	1 (12.5%)	0.527
Central nervous system	6 (4.1%)	1 (4.1%)	>0.999	1 (1.3%)	0 (0.0%)	>0.999
Sin and soft tissue	7 (4.7%)	0 (0.0%)	0.595	4 (5.4%)	0 (0.0%)	>0.999
*Pathogenic bacteria*						
*DTR-PA*	106 (77.6%)	18 (75.0%)	0.806	25 (55.6%)	28 (52.8%)	0.058
Only *CRPA* infection	58 (39.7%)	6 (25.0%)	0.168	29 (39.1%)	1 (12.5%)	0.270
+*CRAB*	47 (32.1%)	12 (50.0%)	0.089	27 (36.4%)	4 (50.0%)	0.715
+*CRKP*	63 (43.1%)	7 (29.1%)	0.197	33 (44.5%)	3 (37.5%)	0.993
+ Other CREs	3 (2.0%)	2 (8.3%)	0.146	1 (1.3%)	2 (25.0%)	**0.024**
Antibiotic regimens						
Treatment course (day)	10.50 (6.50–14.75)	9.50 (7.00–13.25)	0.991	10.00 (7.00–13.75)	7.75 (6.00–11.75)	0.434
Combined antibiotics of *anti-PA*	2.00 (1.00–2.00)	2.00 (1.00–2.00)	0.652	2.00 (1.00–2.00)	1.50 (1.00–2.00)	0.858
Monotherapy	60 (41.1%)	7 (29.1%)	0.268	35 (47.3%)	4 (50.0%)	>0.999
*+ Quinolones*	9 (6.1%)	4 (16.6%)	0.168	5 (6.7%)	1 (12.5%)	0.471
*+ Aminoglycosides*	12 (8.2%)	1 (4.1%)	0.781	8 (10.8%)	0 (0.0%)	>0.999
*+Other β-lactam of anti-PA*	40 (27.4%)	6 (25.0%)	0.806	16 (21.6%)	0 (0.0%)	0.319
*+ Carbapenem*	40 (27.4%)	4 (16.6%)	0.266	16 (21.6%)	3 (37.5%)	0.569
PMB-based regimens	79 (54.1%)	14 (58.3%)	0.700	36 (48.6%)	5 (62.5%)	0.710
AKI	44 (30.1%)	8 (33.3%)	0.753	22 (29.7%)	5 (62.5%)	0.139
Microbiological clearance	63 (43.1%)	9 (37.5%)	0.604	42 (56.7%)	5 (62.5%)	>0.999

Abbreviations are the same as Table 1. Bold font indicates data with significant differences.

**TABLE 10:** COX analysis of factors associated with 30-day all-cause mortality.

Demographics and clinical characteristics	Before PSM			After PSM		
	B	HR (95% CI)	P-value	B	HR (95% CI)	P-value
PMB-based regimens	0.886	2.426 (0.886–6.646)	0.085	0.882	2.416 (0.282–20.723)	0.421
*DTR-PA*	−0.088	0.916 (0.340–2.471)	0.862	0.374	1.453 (0.164–12.879)	0.737
APACHE II	0.707	1.072 (1.032–1.114)	**<0.001**	0.098	1.103 (1.015–1.198)	**0.021**
Sepsis/septic shock	0.994	2.702 (1.115–6.548)	**0.028**	1.961	7.106 (0.744–67.857)	0.088
Vasoactive drugs	1.193	3.298 (0.909–11.965)	0.070	−0.631	0.532 (0.060–4.698)	0.570
Other CREs infection	1.492	4.488 (0.925–21.383)	0.062	3.710	40.849 (3.323–502.170)	**0.004**
Microbiological clearance	0.314	1.368 (0.521–3.596)	0.524	−0.001	0.999 (0.150–6.663)	0.999
AKI	0.072	1.075 (0.446–2.593)	0.872	0.724	2.063 (0.362–11.775)	0.415

The multivariate analysis model included all variables with p < 0.05 from the univariate analysis of data before and after PSM, as well as variables fixed based on CAZ/AVI, AKI, microbiological clearance, DTR-PA.Bold font indicates data with significant differences.

## 4 Discussion

This study, based on real-world multicentre data, aims to investigate the efficacy and safety of PMB-based regimens and CAZ/AVI-based regimens in the treatment of *CRPA* infections. It was the first study to examine the clearance rate of *CRPA*, the incidence of AKI, and the influencing factors associated with these two treatment regimens. Both 1:1 PSM and multivariable analyses independently demonstrated significantly superior microbiological clearance of *CRPA* with CAZ/AVI-based regimens versus PMB-based regimens. However, neither analytical approach revealed statistically significant differences in clinical efficacy, AKI incidence, or 30-day all-cause mortality between treatment groups.

In terms of clinical efficacy, there was no statistically significant difference in clinical success rates between CAZ/AVI-based and PMB-based regimens. The overall clinical success rate for CRPA infections was consistent with previously reported data (51.2% vs. 63.1%) (Xu et al., 2024). Before PSM, multivariable regression analysis identified *DTR-PA* infections and vasopressor requirements as independent risk factors for treatment failure in *CRPA* infections. *PA* multifaceted resistance mechanisms (efflux pump overexpression/porin loss/β-lactamase production) pose therapeutic challenges, and *DTR-PA* had also been proposed (Cosentino et al., 2023). Notably, *PA* virulence determinants (exotoxin A, type III secretion system) have been demonstrated to be critical drivers of ventilator-associated pneumonia (VAP) progression, with severe cases predisposing to multiorgan dysfunction and consequent escalation of vasopressor dependency (Alonso et al., 2020). This finding underscores the imperative for comprehensive analysis of *DTR-PA* virulence determinants, particularly given their demonstrated role in treatment failure and clinical deterioration.

The *CRPA* clearance rates observed in this study were higher compared to previous studies (CAZ/AVI: 59.7% vs. 45.1%; PMB: 28.0% vs. 14.3%), possibly due to the exclusion of patients receiving low-dose PMB (Chen et al., 2022). In regimens of medication choices, both the *CRPA* clearance rates for CAZ/AVI monotherapy and combination therapy were superior to the PMB-based regimens. Multifactorial analysis of *CRPA* microbiological efficacy also shows that the CAZ/AVI regimen was an independent predictor for *CRPA* clearance, consistent with guidelines recommending CAZ/AVI as the preferred treatment for *CRPA* infections (Tamma et al., 2024, Pulmonary Infection Assembly of Chinese Thoracic, 2022; Tamma et al., 2022). Furthermore, multivariable analysis after PSM indicated that a higher number of combined anti-*PA* antibiotics was associated with improved *CRPA* clearance rates, indirectly suggesting the potential benefits of appropriate combination therapy in the management of *CRPA* infections.

However, when *in vitro* susceptibility results indicated sensitivity to first-line drugs like CAZ/AVI, combination antibiotic therapy was not recommended (Tamma et al., 2024; Pulmonary Infection Assembly of Chinese Thoracic, 2022). Nevertheless, the total microbiological clearance rate for PMB treatment of *CRPA* infections with a median duration of 10.0 days was only 28.0% (monotherapy 21.4% vs. combination 30.8%), suggesting that combination therapy was a viable option for increasing the *CRPA* clearance rate with PMB-based regimens (Wang et al., 2022). Intriguingly, 25% of the patients in our cohort received carbapenems as part of their treatment regimen. Although *in vitro* studies had demonstrated synergistic effects of meropenem and colistin against *CRPA* (Gunalan et al., 2021), the *in vivo* and *in vitro* efficacy of CAZ/AVI combined with carbapenem antibiotics for CRPA infections had not yet been reported. In our study, nine patients received CAZ/AVI in combination with carbapenems.

In hospitalized patients, the occurrence of AKI was mainly related to sepsis, hypotension, and medications. The management of nephrotoxic drugs was one of the main strategies for AKI management (Kellum et al., 2021). Real-world data suggested that the incidence of PMB-induced AKI in the Chinese population is around 33.5%, mainly related to loading dose, concomitant nephrotoxic drugs, and baseline creatinine levels (Chang et al., 2022). Our study results shown that the incidence of AKI in the PMB group was similar to previous studies on PMB-related AKI (35.5% vs. 33.5%) (Chang et al., 2022). CAZ/AVI was generally well-tolerated, with most adverse events being mild to moderate. The incidence of AKI in real-world data for CAZ/AVI ranges from 10% to 38% (Shi et al., 2024; Feldman et al., 2022). Our study results suggested that the incidence of AKI in patients treated with CAZ/AVI is 24.7%. The incidence of AKI was higher following PMB treatment compared to CAZ/AVI, although the difference was not statistically significant (35.5% vs. 24.7%). This finding was consistent with previous studies (Chen et al., 2024).

However, multivariate analysis demonstrated that, prior to PSM, the risk of AKI was significantly higher with the PMB regimen compared to the CAZ/AVI regimen. After PSM, this difference was no longer statistically significant. This attenuation of significance might be attributable to the relatively preserved baseline renal function among the *CRPA*-infected patients included in our study, and given that baseline renal function constitutes the primary independent predictor of PMB-associated AKI(Wu et al., 2022). Specifically, the median baseline serum creatinine levels were 82.3 μmol/L after PSM, and the median creatinine clearance was 74.6 mL/min after PSM. Moreover, there were no significant differences in baseline renal function between the PMB and CAZ/AVI cohorts.

In our PMB cohort study, 91.4% of patients had *CRPA* lung infection. Adherence to the recommended PMB dosage was suboptimal, with only 73.1% of patients received the prescribed dose of 50 mg q12h, and merely 49.5% received a loading dose. Current PMB dosing guidelines suggested a loading dose ranging from 2.0–2.5 mg/kg and a maintenance dose of 1.25–1.5 mg/kg infused every 12 h (Tsuji et al., 2019). Nebulized PMB was proposed as a potential alternative to intravenous administration in ventilator-associated pneumonia patients, considering nephrotoxicity concerns (Shi et al., 2023). However, loading doses of PMB were independently associated with nephrotoxicity risks, and monitoring PMB blood concentrations was crucial in critically ill patients (Chang et al., 2022; Nation et al., 2017). Multi-centre studies suggested that combined nebulized PMB did not significantly impact the cure rate of ventilator-associated pneumonia in CRGNB-infected patients (Liu et al., 2022).

In the case of CAZ/AVI, the recommended dosage for adult patients with a CCR >50 mL/min was 2.5 g q8h administered as a continuous intravenous infusion over 2 h (Das et al., 2019). Adjustments to the CAZ/AVI dosage were warranted for patients with a CCR≤50 mL/min (Das et al., 2019). Blood concentrations of CAZ/AVI differ based on renal function status, with dosing adjustments required for patients with renal impairment (Kang et al., 2021; Teng et al., 2022). The 2.5 g q8h regimen was deemed feasible for critically ill patients with *MDR-PA* lung infections undergoing CRRT (Soukup et al., 2019). Patients with augmented renal clearance (CCR>130 mL/min) might necessitate higher CAZ/AVI dosages to achieve PK/PD targets (Dai et al., 2021). In our study of CAZ/AVI cohort patients, 22.1% had pre-existing renal insufficiency. Most patients (85.7%) adhered to the recommended dosing regimen, while 9.1% required dose adjustments during treatment.

In the primary endpoints of this study, there was no difference in the 30-day all-cause mortality before and after PSM. We compared the characteristics of survivors and non-survivors at 30 days and explored potential independent influencing factors through multivariate analysis. In the univariate analysis, AKI and bacterial clearance rate did not show significant differences between the survival and non-survival groups. Notably, our study revealed a higher prevalence of *DTR-PA* infections compared to previous reports (72.9% vs. 34%–38%) (Dong et al., 2025; Yuan et al., 2023); interestingly, in our study, *DTR-PA* cases demonstrated a 30-day all-cause mortality rate of 14.5%, numerically lower than the 43% rate historically reported (Yuan et al., 2023). This discrepancy may be attributable to the inclusion of cases exclusively from high-volume tertiary care centres in China, which typically have advanced antimicrobial stewardship programs and critical care capabilities.

The multivariate COX regression analysis revealed that APACHE II score were independent risk factors for 30-day all-cause mortality. In previous studies treating *CRPA* or *DTR-PA* infections with CAZ/AVI (Xu et al., 2024), the APACHE II score and sepsis/sepsis shock at the onset of infection shown significant differences between the survival and non-survival groups, which seems like our findings. In another study comparing PMB with CAZ/AVI for *CRPA* treatment (Chen et al., 2022), sepsis shock was also confirmed as an independent predictor for 30-day all-cause mortality, and the CAZ/AVI regimen was an independent predictor for 30-day survival compared to the PMB regimen. However, in our study, the CAZ/AVI regimens and *DTR-PA* infections did not significantly impact 30-day survival rates compared to the PMB regimen.

This study has several limitations. Firstly, the observational design introduced immortal-time bias, as patients had to survive long enough to receive CAZ/AVI or PMB therapy. This bias was compounded by the fact that newer antibiotics were often reserved for resistant cases, potentially excluding patients who died prematurely. Additionally, the requirement for 72 h of effective therapy further exacerbated this bias. Secondly, the inclusion of polymicrobial infections, particularly those involving *CRKP* (41.2%), represented a limitation. Although *CRKP* presence was matched, data on the susceptibility of co-pathogens to CAZ/AVI and PMB were not included. Thirdly, as a retrospective study, not all patients underwent rechecking for *CRPA* colonization at the end of drug therapy in real-world clinical settings, which may have impacted our results. Fourthly, we were unable to ascertain the proportion of carbapenemase-producing strains among the *CRPA* isolates, as resistance mechanisms were not characterized for any included strains. Lastly, our sample size was limited, and the varying medical standards across different centres could have influenced the results. Furthermore, we did not categorize *CRPA* based on genotype or biofilm formation status, which necessitates further evaluation of the efficacy of different resistance mechanisms.

## 5 Conclusion

In conclusion, for the treatment of *CRPA* infection, CAZ/AVI demonstrates superior efficacy in microbiological clearance of *CRPA* compared to PMB. However, the clinical efficacy was comparable between the two treatment regimens. These findings warrant validation through large-scale prospective studies to further elucidate the comparative effectiveness of these antimicrobial agents.

## Data Availability

The original contributions presented in the study are included in the article/Supplementary Material, further inquiries can be directed to the corresponding author.
