# Supplementary material for: The efficacy and safety of ceftazidime/avibactam or polymyxin B based regimens for carbapenem-resistant Pseudomonas aeruginosa infection: a multicenter real-world and propensity score-matched study
Source: Front Pharmacol. 2025 Mar 31;16:1533952. doi: 10.3389/fphar.2025.1533952 (PMC11994704; doi:10.3389/fphar.2025.1533952)
Supplement: Supplementary file 1 [file DataSheet1.zip › Supplementary file 4.DOCX]

**Supplementary file 4**

**Comparative Analysis of Clinical Outcomes Between Combination Therapy and Monotherapy**

| **Antibiotic regimens** | **PMB-based regimens** | | | | **CAZ/AVI-based regimens** | | | |
| --- | --- | --- | --- | --- | --- | --- | --- | --- |
|  | **Total (N=93)** | **Combination therapy (N=65)** | **Monotherapy  (N=28)** | **P-value** | **Total (N=77)** | **Combination therapy (N=38)** | **Monotherapy (N=39)** | **P-value** |
| Clinical efficacy | 45(48.4%) | 35(53.8%) | 10(35.7%) | 0.108 | 42(54.5%) | 21(55.3%) | 21(53.8%) | 0.901 |
| 7-day microbiological clearance | 15(16.1%) | 11(16.9%) | 4(14.3%) | 0.992 | 28(36.4%) | 17(44.7%) | 11(28.2%) | 0.132 |
| Microbiological clearance | 26(28.0%) | 20(30.8%) | 6(21.4%) | 0.357 | 46(59.7%) | 24(63.2%) | 22(23.3%) | 0.546 |
| 30-day mortality | 14(15.1%) | 10(15.4%) | 4(14.3%) | ＞0.999 | 10(13.0%) | 7(18.4%) | 3(7.7%) | 0.289 |
| AKI | 33(35.5%) | 21(32.3%) | 12(42.9%) | 0.329 | 19(24.8%) | 12(31.6%) | 7(17.9%) | 0.165 |
