# Supplementary material for: The efficacy and safety of ceftazidime/avibactam or polymyxin B based regimens for carbapenem-resistant Pseudomonas aeruginosa infection: a multicenter real-world and propensity score-matched study
Source: Front Pharmacol. 2025 Mar 31;16:1533952. doi: 10.3389/fphar.2025.1533952 (PMC11994704; doi:10.3389/fphar.2025.1533952)
Supplement: Supplementary file 1 [file DataSheet1.zip › Supplementary file 3.DOCX]

Supplementary file 3

Antibiotic regimens for patients with polymicrobial infections.

| **Antibiotic regimens** | **All**  **N=170** | **PMB**  **N=93** | **CAZ/AVI**  **N=77** | **p-value** |
| --- | --- | --- | --- | --- |
| **Only *CRPA* infection** | **N=64** | **N=38** | **N=26** |  |
| Combined antibiotics of anti-PA | 2.0(1.0-3.0) | 2.0(2.0-3.0) | 2.0(1.0-2.0) | **0.013** |
| Monotherapy | 17(26.6%) | 5(13.2%) | 12(46.2%) | **0.003** |
| + Quinolones | 6(4.7%) | 3(7.9%) | 3(11.5%) | 0.680 |
| + Aminoglycosides | 6(9.4%) | 4(10.5%) | 2(3.1%) | ＞0.999 |
| +Other β-lactam of anti-PA | 19(29.7%) | 15(39.5%) | 4(15.4%) | 0.073 |
| +Carbapenem | 26(40.6%) | 20(52.6%) | 6(23.1%) | **0.018** |
| +Tigecycline | 0 | 0 | 0 | - |
| **Co-infection of *CRPA* and *CRKP*** | **N=70** | **N=27** | **N=43** |  |
| Combined antibiotics of anti-PA | 2.0(1.0-2.0) | 2.0(1.0-2.0) | 2.0(1.0-2.0) | 0.589 |
| Monotherapy | 32(45.7%) | 11(40.7%) | 21(48.8%) | 0.508 |
| + Quinolones | 5(7.1%) | 1(3.7%) | 4(9.3%) | 0.642 |
| + Aminoglycosides | 8(11.4%) | 3(11.1%) | 5(11.6%) | ＞0.999 |
| +Other β-lactam of anti-PA | 17(24.3%) | 8(29.6%) | 9(20.9%) | 0.409 |
| +Carbapenem | 11(15.7%) | 8(29.6%) | 3(7.0%) | **0.028** |
| +Tigecycline | 11(22.0%) | 7(25.9%) | 4(17.4%) | 0.701 |
| **Co-infection of *CRPA* and *CRAB*** | **N=59** | **N=37** | **N=22** |  |
| Combined antibiotics of anti-PA | 1.0(1.0-2.0) | 2.0(1.0-2.0) | 1.0(1.0-2.0) | **0.038** |
| Monotherapy | 30(50.8%) | 15(40.5%) | 15(68.2%) | **0.040** |
| + Quinolones | 3(5.1%) | 3(8.1%) | 0(0.0%) | 0.286 |
| + Aminoglycosides | 2(3.4%) | 1(2.7%) | 1(4.5%) | ＞0.999 |
| +Other β-lactam of anti-PA | 14(23.7%) | 13(35.1%) | 1(4.5%) | **0.019** |
| +Carbapenem | 11(18.6%) | 10.0(27.0%) | 1(4.5%) | 0.072 |
| +Tigecycline | 8(13.6%) | 3(8.1%) | 5(22.7%) | 0.148 |

Other *CREs* infection cases were not included in the statistics due to their limited number. Among the *CRPA+CRKP* and *CRPA+CRAB* groups, there were eight cases that were simultaneously infected with *CRPA*, *CRKP*, and *CRAB*. Additionally, the data after propensity score matching (PSM) were not subjected to stratified statistical analysis due to the limited number of cases.
