# Supplementary figures and images for: The efficacy and safety of ceftazidime/avibactam or polymyxin B based regimens for carbapenem-resistant Pseudomonas aeruginosa infection: a multicenter real-world and propensity score-matched study
Source: Front Pharmacol. 2025 Mar 31;16:1533952. doi: 10.3389/fphar.2025.1533952 (PMC11994704; doi:10.3389/fphar.2025.1533952)

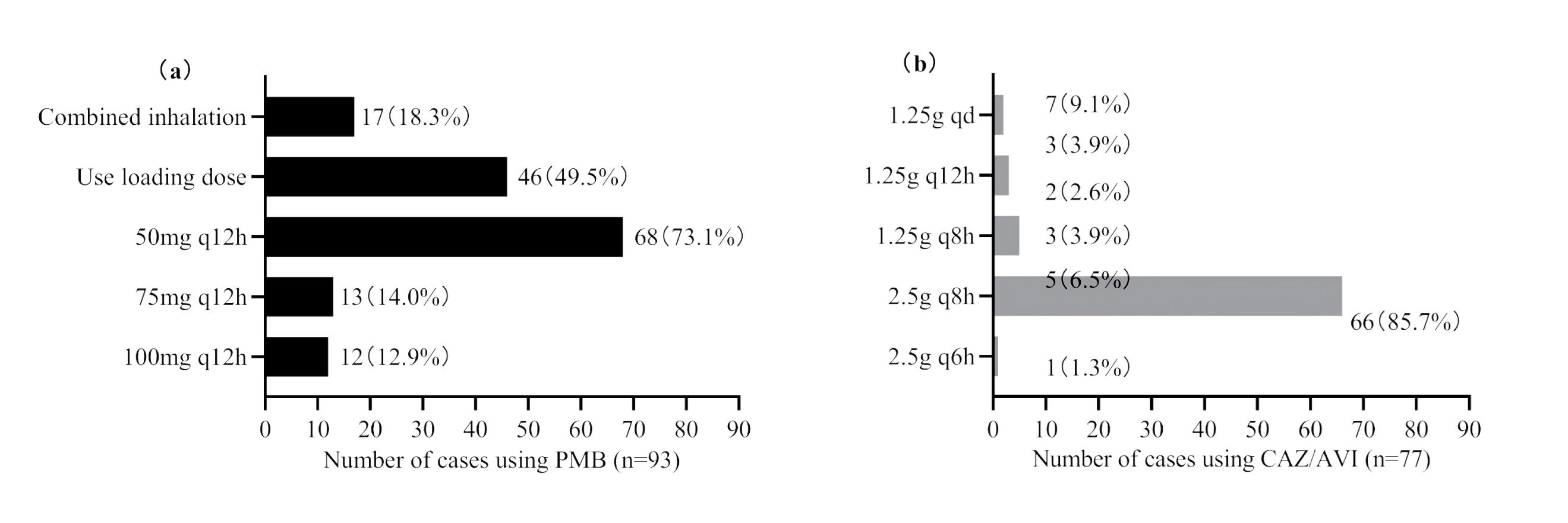

Supplement: Supplementary file 1 [file DataSheet1.zip › Supplementary file 1.JPEG]

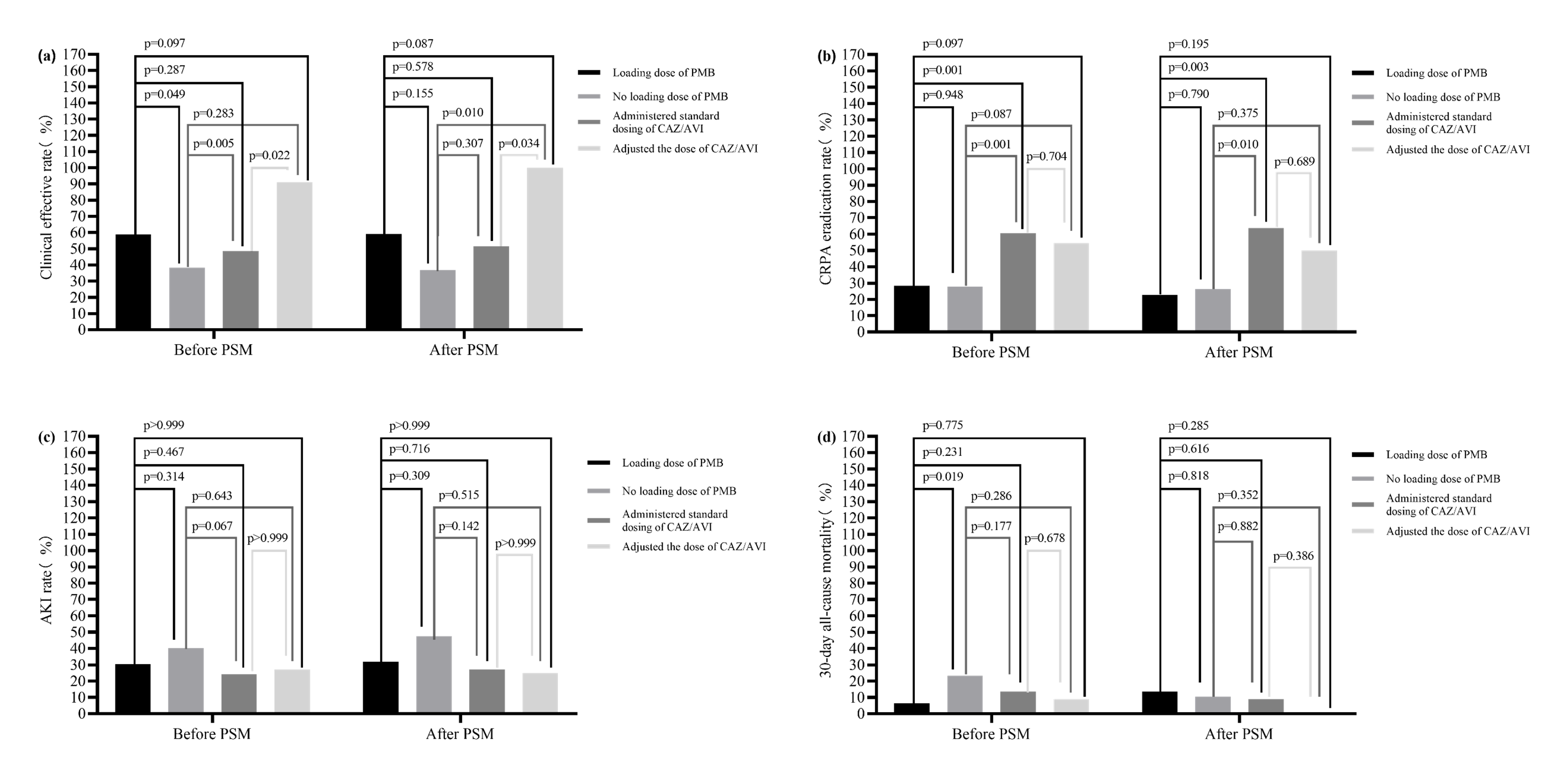

Supplement: Supplementary file 1 [file DataSheet1.zip › Supplementary file 2.TIF]

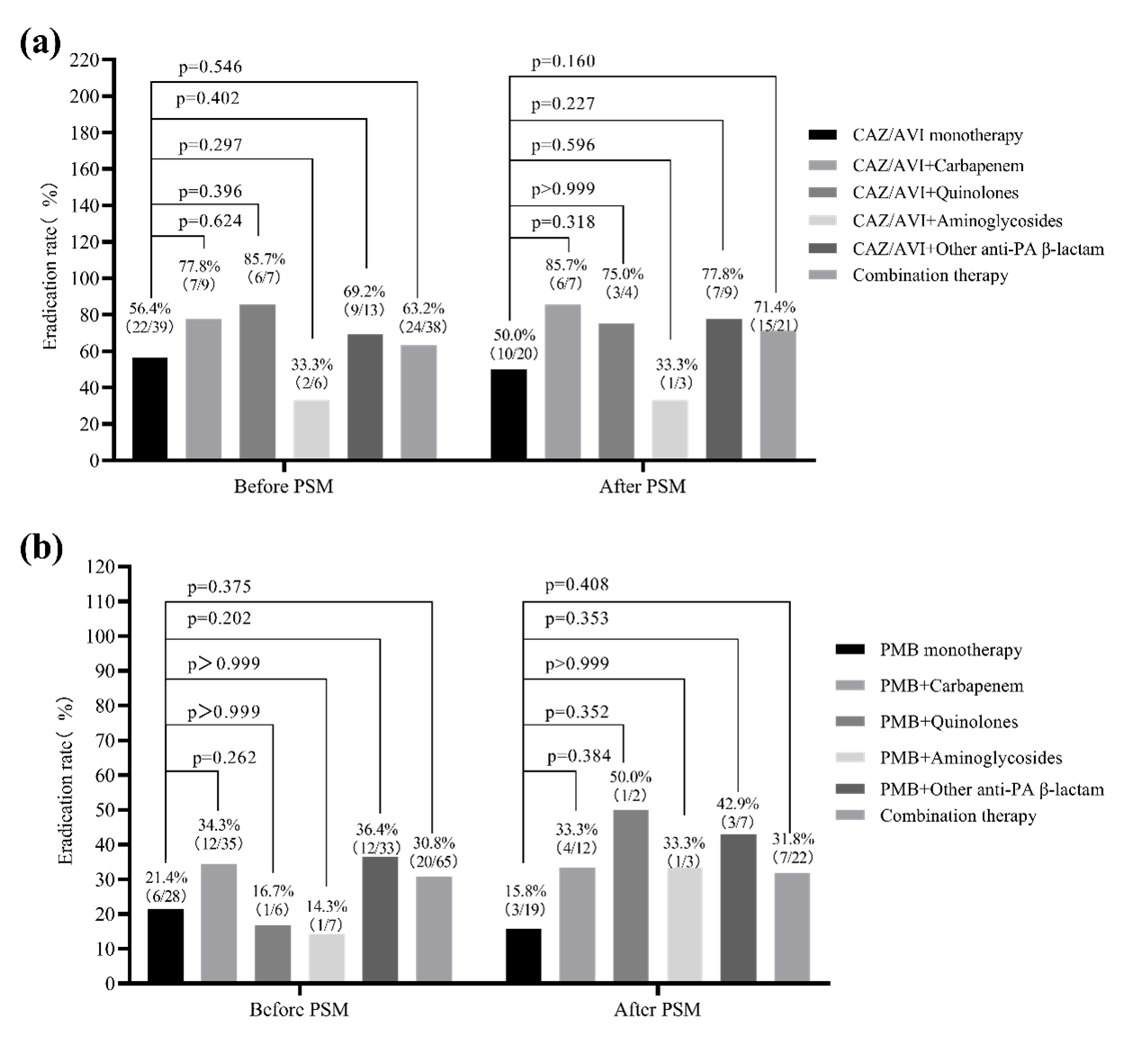

Supplement: Supplementary file 1 [file DataSheet1.zip › Supplementary file 5.TIF]
